# Structure and kinase activity of bacterial cell cycle regulator CcrZ

**DOI:** 10.1371/journal.pgen.1010196

**Published:** 2022-05-16

**Authors:** Katherine J. Wozniak, Peter E. Burby, Jayakrishnan Nandakumar, Lyle A. Simmons

**Affiliations:** Department of Molecular, Cellular, and Developmental Biology, University of Michigan, Ann Arbor, Michigan, United States of America; Max Planck Institute for Terrestrial Microbiology: Max-Planck-Institut fur terrestrische Mikrobiologie, GERMANY

## Abstract

CcrZ is a recently discovered cell cycle regulator that connects DNA replication initiation with cell division in pneumococci and may have a similar function in related bacteria. CcrZ is also annotated as a putative kinase, suggesting that CcrZ homologs could represent a novel family of bacterial kinase-dependent cell cycle regulators. Here, we investigate the CcrZ homolog in *Bacillus subtilis* and show that cells lacking *ccrZ* are sensitive to a broad range of DNA damage. We demonstrate that increased expression of *ccrZ* results in over-initiation of DNA replication. In addition, increased expression of CcrZ activates the DNA damage response. Using sensitivity to DNA damage as a proxy, we show that the negative regulator for replication initiation (*yabA*) and *ccrZ* function in the same pathway. We show that CcrZ interacts with replication initiation proteins DnaA and DnaB, further suggesting that CcrZ is important for replication timing. To understand how CcrZ functions, we solved the crystal structure bound to AMP-PNP to 2.6 Å resolution. The CcrZ structure most closely resembles choline kinases, consisting of a bilobal structure with a cleft between the two lobes for binding ATP and substrate. Inspection of the structure reveals a major restructuring of the substrate-binding site of CcrZ relative to the choline-binding pocket of choline kinases, consistent with our inability to detect activity with choline for this protein. Instead, CcrZ shows activity on D-ribose and 2-deoxy-D-ribose, indicating adaptation of the choline kinase fold in CcrZ to phosphorylate a novel substrate. We show that integrity of the kinase active site is required for ATPase activity *in vitro* and for function *in vivo*. This work provides structural, biochemical, and functional insight into a newly identified, and conserved group of bacterial kinases that regulate DNA replication initiation.

## Author summary

Some bacteria enlist kinases to regulate cell cycle progression and developmental checkpoints. CcrZ is a recently identified cell cycle regulator and putative kinase showing similarity to choline kinases. We identified the *ccrZ* gene in *Bacillus subtilis* using a forward genetic approach with the *ccrZ* deletion conferring sensitivity to a broad range of DNA damage. We show that induced expression of CcrZ causes over-initiation of DNA replication, and we provide evidence for an interaction between CcrZ and replication initiation proteins DnaA and DnaB. We find that integrity of the kinase active site is required for function *in vivo*. To gain insight into kinase function, we solved the crystal structure of *B*. *subtilis* CcrZ bound to AMP-PNP. The structure demonstrates that CcrZ evolved from a choline kinase with a bilobed structure and a larger inter-lobe cleft relative to choline kinases. The larger inter-lobe cleft could help accommodate larger substrates than choline. As expected from the structure, CcrZ does not have activity on choline, but instead shows activity on ribose and 2 deoxy-ribose. With our results, we propose that CcrZ regulates replication initiation through phosphorylation of a small molecule and interaction with replication initiation proteins.

## Introduction

Bacteria need to faithfully replicate their DNA and properly segregate new chromosomes into daughter cells to maintain genome integrity and cell viability. Such an effort requires that all cells coordinate DNA replication with cell division and repair any damage that is encountered during the process [1,2]. To help ensure each of these processes occurs at the correct time during the cell cycle, regulatory mechanisms control replication initiation, cell division, and cell cycle progression when DNA problems are detected [3,4].

DNA replication consists of three steps: initiation, elongation, and termination. Regulation often occurs during initiation by modulating the activity of the initiator protein DnaA (for review [5]). When DnaA is bound to ATP, it binds DnaA-boxes at *oriC* [6]. DnaA-ATP then initiates melting of the AT-rich region at the origin [7]. In *Bacillus subtilis*, DnaA along with essential adaptors DnaB and DnaD load the replicative helicase, allowing for bidirectional fork movement [8,9]. Overexpression or depletion of *dnaA* has been shown to elongate cells and lead to anucleate cell formation [10]. YabA negatively regulates DnaA during vegetative growth and has been shown to bind both DnaA and DnaN (β clamp)[11,12]. Importantly, cells lacking *yabA* exhibit over-initiation phenotypes and asynchronous DNA replication, underscoring the need for YabA-dependent regulation of initiation timing [13]. YabA has been shown to bind domain III of DnaA and inhibit cooperative DnaA binding to the origin [9,14,15]. During *B*. *subtilis* sporulation, DnaA is also a target for negative regulation by the protein SirA to prevent new initiation events from occurring, maintaining the genome copy number at two chromosomes during spore development [16]. SirA has been shown to interact with DnaA and prevent replication initiation in part by binding to a site on domain I which overlaps with the DnaD binding site on DnaA [9,17]. Therefore, in *B*. *subtilis*, DnaA is targeted for negative regulation by YabA during normal growth and SirA during sporulation to ensure proper levels of DNA replication initiation.

Bacteria frequently experience DNA damage during normal growth, subsequently inhibiting cell division allowing for the repair of damaged chromosomes [18]. Single stranded DNA resulting from stalled replication or DNA damage is bound by RecA [for review [19,20]. Once a threshold of RecA/ssDNA is reached, the transcriptional repressor LexA undergoes autocleavage causing derepression of the ~63 genes involved in the *B*. *subtilis* DNA damage response (SOS) [21,22]. The *yneA* gene is regulated by LexA and is highly expressed in response to DNA damage [18]. YneA inhibits cell division by binding peptidoglycan and interfering with the interaction between late arriving divisome proteins [23]. In addition to the YneA-enforced DNA damage checkpoint, *B*. *subtilis* cells deplete the essential cell division protein FtsL via DnaA in response to perturbations to replication fork progression [24]. Therefore, in *B*. *subtilis* more than one mechanism exists to regulate cell division when genome integrity is compromised. In the case of FtsL, depletion is regulated by DnaA providing a connection between replication initiation and the regulation of cell division [24].

In addition to these processes, a “failsafe mechanism” was described in *B*. *subtilis* that links DNA replication initiation to cell division [25]. This work showed that when cell division is blocked, DNA replication initiation is also blocked, indicating that replication initiation and cell division are coupled [25]. Although the mechanism is unclear, cells were shown to enter a quiescent state where they were metabolically active but failed to re-enter the cell cycle [25]. Recently in *Streptococcus pneumoniae*, a new cell cycle regulator was described to directly coordinate DNA replication initiation with cell division [26]. This conserved Cell Cycle Regulator protein interacting with FtsZ (CcrZ) was shown to interact with FtsZ, a tubulin homolog required for Z-ring formation and cytokinesis [26]. CcrZ in *S*. *pneumoniae* has also been shown to regulate replication initiation through the DnaA-dependent origin *oriC* [26]. *S*. *pneumoniae* CcrZ colocalized with FtsZ and DnaA bound to *oriC in vivo* [26]. Deletion or depletion of *ccrZ* in *S*. *pneumoniae* led to aberrant cell division, anucleate cells, and under-replication, further suggesting that CcrZ couples DNA replication initiation with cell division [26]. The *ccrZ* gene is highly conserved among bacteria in the phylum Firmicutes [26]. Studies of the *B*. *subtilis* homolog YtmP show that deletion of *ytmP* leads to under initiation without an observable effect on cell division [26]. Another study identified mutations in *ytmP* that suppressed a conditional lethal phenotype of the *dnaA1(ts)* Δ*yabA* double mutant, further indicating that YtmP provides a regulatory contribution to DNA replication initiation in *B*. *subtilis* [27]. Thus, CcrZ/YtmP could have a conserved role in regulating DnaA-dependent replication initiation in a broad group of bacteria including several human pathogens. CcrZ is annotated as a putative choline kinase, although structural and biochemical data have yet to be established for CcrZ from any organism.

We previously conducted a saturating transposon screen in *B*. *subtilis* against several DNA damaging agents including mitomycin C [28]. This work initially identified *ccrZ* as a putative phosphotransferase that conferred sensitivity to DNA damage when gene function was lost [28]. Here, we show that deletion of *ccrZ* results in sensitivity to a wide range of DNA damage. We show that the amount of *ccrZ* in the cell is important for maintaining WT growth under DNA damaging conditions. We find that overexpression of *ccrZ* results in over-initiation of DNA replication and that CcrZ interacts with initiation proteins DnaA and DnaB. To gain further insight into CcrZ, we solved the crystal structure bound to AMP-PNP and demonstrate that CcrZ evolved from a choline kinase to phosphorylate a different substrate. ATPase assays with purified CcrZ show activity on D-ribose and 2-deoxy-D-ribose, suggesting that CcrZ could phosphorylate a metabolite or small molecule. Importantly, ablation of the kinase active site results in a mutant unable to complement the Δ*ccrZ* phenotype, demonstrating that kinase activity is required for function *in vivo*. Our functional and structural characterization provides novel insights into this new family of conserved bacterial cell cycle regulators.

## Results

### Δ*ccrZ* sensitizes *B*. *subtilis* to DNA damage and *ccrZ* overexpression causes cell elongation

**Fig 1:**
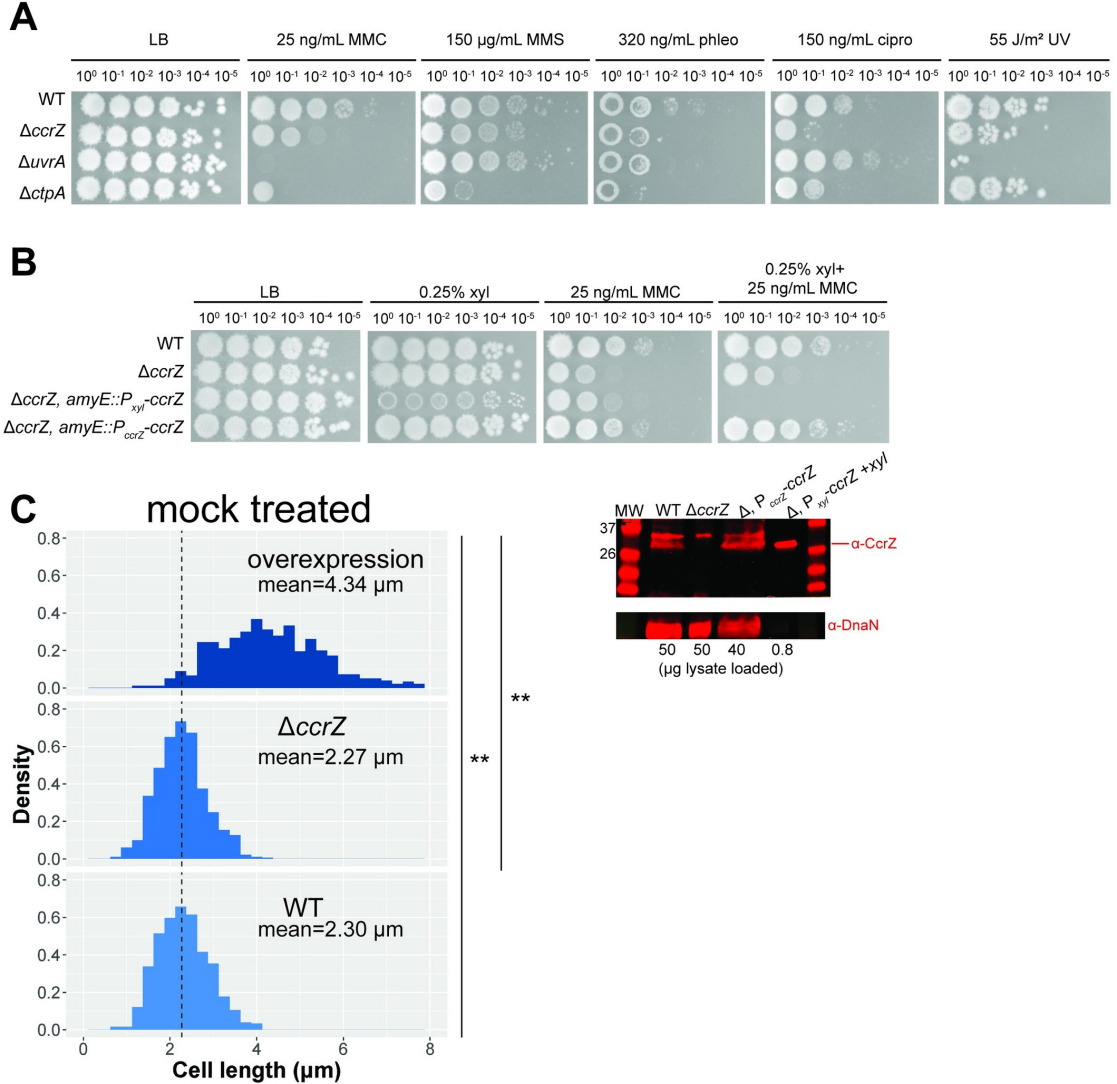
Investigation of Δ*ccrZ* sensitivity to DNA damage and effect of deletion and overexpression on cell length. **(A)** Spot titer assay showing Δ*ccrZ* is sensitive to mitomycin C (MMC), methyl methanesulfonate (MMS), ciprofloxacin (cipro), phleomycin (phleo), and UV. **(B)** Complementation of Δ*ccrZ* achieved using the *ccrZ* endogenous promoter (522 bp upstream of coding sequence) from *amyE*. **(C)** Left: overexpression of *ccrZ* significantly elongates cells while deletion does not change cell length. n = 724 cells for overexpression (Δ*ccrZ*, *amyE*::P_*xyl*_*-ccrZ-chl* with 0.1% xylose), n = 723 cells for Δ*ccrZ*, and n = 725 cells for WT. Dashed line indicates WT mean length (2.30 μm). Wilcoxon rank-sum tests were performed on cell lengths of all three genotypes; **p = 2.2E^-16^. Right: Western blot detecting CcrZ and a loading control, DnaN, for WT, deletion, endogenous promoter driven *ccrZ* expression, and *ccrZ* overexpression from a xylose-inducible promoter. Amount of lysate loaded (in μg) indicated below each lane.

We initially discovered the gene *ytmP/ccrZ* in a Tn-seq screen for genes that confer sensitivity to DNA damage [28]. For consistency with recent literature, we will refer to *B*. *subtilis ytmP* hereafter as *ccrZ* [26]. To validate the sensitivity to mitomycin C (MMC) of *ccrZ*::*Tn* from the Tn-seq screen, we created a clean deletion using CRISPR/Cas9 (Δ*ccrZ*) [29]. We tested *B*. *subtilis* cells with Δ*ccrZ* on a variety of DNA damaging agents to determine if the sensitivity to MMC was specific or indicative of a general sensitivity to genotoxic stress. As controls, we included Δ*uvrA*, involved in nucleotide excision repair of bulky DNA adducts [30,31], and Δ*ctpA*, a protease responsible for degrading the DNA damage checkpoint protein YneA [28]. Deletion of *ccrZ* renders cells sensitive to the crosslinking agent MMC [32], the methylating agent methyl methanesulfonate (MMS) [33], the topoisomerase inhibitor ciprofloxacin [34], and the break inducing peptide phleomycin [35] **(Fig 1A).** Sensitivity to such a broad range of DNA damaging agents was not simply a result of an increase in cellular permeability because Δ*ccrZ* also conferred sensitivity to UV irradiation **(Fig 1A)**.

Our prior sequencing, genome assembly and genome annotation predicts 4 additional amino acids to the CcrZ N-terminus for PY79 relative to *S*. *pneumoniae* CcrZ [36] **(S5A Fig)**. Using this construct, we tested a complementing allele to restore growth to WT in the deletion background. We were unable to achieve full complementation of the Δ*ccrZ* using a xylose-inducible promoter driving *ccrZ* expression from an ectopic locus, despite testing various concentrations of xylose **(Fig 1B)**. Importantly, xylose induction severely sensitized cells to DNA damage **(Fig 1B)**. With this result we suggest that the amount of CcrZ in the cell is important for proper function. We were able to fully complement Δ*ccrZ* after cloning the 522 bp region upstream of the open reading frame to drive *ccrZ* expression from an ectopic locus **(Fig 1B)**. Using anti-CcrZ antiserum developed against purified CcrZ protein, we detect full length CcrZ using a Western blot with WT, the endogenous promoter driving *ccrZ*, and overexpressed *ccrZ* following xylose induction **(Fig 1C)**. We also detect a higher molecular weight cross-reacting band present in lysates including the deletion, confirming that the lower band corresponds to CcrZ. The cross-reacting protein is not visible in the xylose induced lane because we loaded considerably less lysate to clearly detect the size of xylose induced CcrZ. For this reason, DnaN is also not detected as a loading control for the xylose induced CcrZ lane. We conclude that the upstream cloned region does contain the endogenous promoter and that each *ccrZ* construct used yields the same size protein as determined by Western blot. Previously, *S*. *pneumoniae* was shown to produce anucleate cells and aberrant septa in a Δ*ccrZ* background [26]. Using single cell microscopy with FM4-64, we observed no statistically significant difference between WT cell lengths and Δ*ccrZ B*. *subtilis* cells **(Fig 1C)** as suggested previously [26]. In contrast, when we overexpressed *ccrZ* using a xylose-inducible promoter, we observed severe cell elongation **(Figs 1C and S1A)**, again indicating that the amount of CcrZ present in the cell is important for maintaining appropriate cell length and genome integrity.

### Overexpression of CcrZ elicits the SOS response and alters replication initiation

**Fig 2:**
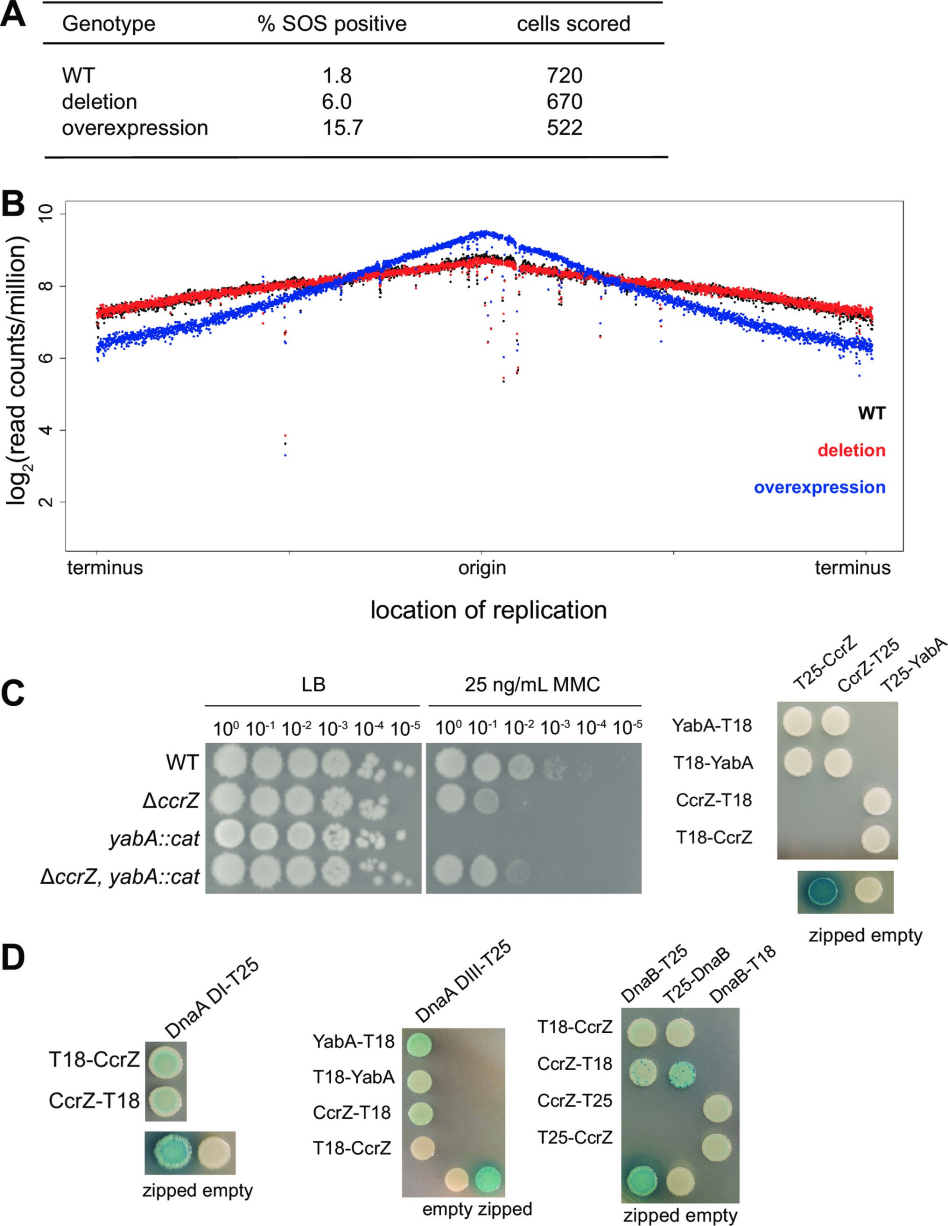
Assessment of *ccrZ* in DNA replication initiation. **(A)** Quantification of % single cells with TagC-GFP as a measure of % SOS induction. p-value (WT vs. deletion) = 9.39x10^-3^, p-value (WT vs. overexpression) = 2.40x10^-4^. **(B)** Averaged whole genome resequencing from three biological replicates of WT (black), Δ*ccrZ* (red), and *ccrZ* overexpression (blue) cells. Log_2_-fold abundance was plotted against chromosome location. **(C)** Spot titer assay to determine genetic relationship between *ccrZ* and *yabA* on MMC (left). Bacterial two-hybrid assay testing for interaction between YabA and CcrZ (right). Three biological replicates were performed before producing the representative image. **(D)** Bacterial two-hybrids showing interaction between CcrZ and DnaA domain I, domain III, and DnaB. Three biological replicates were performed before producing the representative image.

Because Δ*ccrZ c*onfers sensitivity to a broad range of DNA damage we asked if Δ*ccrZ* or overexpression of *ccrZ* influence induction of the DNA damage response (SOS). We quantified the percentage of single cells containing TagC-GFP fluorescence as a reporter because *tagC* is one of the most highly expressed SOS genes in *B*. *subtilis* [37,38]. We found significant SOS induction in cells lacking *ccrZ* compared to WT with ~6% of Δ*ccrZ B*. *subtilis* cells showing induction **(Figs 2A and S1B)**. When *ccrZ* is overexpressed, we observe SOS-induction in ~15.7% of cells, an 8-fold increase over WT **(Figs 2A and S1B)**. These findings, in addition to the severe cell elongation measured during *ccrZ* overexpression, suggest that accumulation of CcrZ in the cell is affecting genome maintenance. Since ectopically induced expression of CcrZ results in a phenotype, we asked if the amount of native CcrZ protein is increased by DNA damage. We performed a Western blot of WT cells treated with MMC or a mock control **(S1C Fig)**. We did not observe a difference in CcrZ protein accumulation following MMC treatment, indicating that native CcrZ expression is not DNA damage inducible **(S1C Fig)**.

Prior work measured the origin/terminus ratio in *B*. *subtilis* showing that Δ*ccrZ* cells under-initiate DNA replication, compared to WT [26,27]. We asked if overexpression of *ccrZ* could result in premature replication initiation leading SOS induction and cell elongation. To understand the effect of Δ*ccrZ* and *ccrZ* overexpression on DNA replication, we performed whole genome re-sequencing for WT, Δ*ccrZ*, and *ccrZ* overexpression to measure replication across the chromosome for averaged triplicates **(Fig 2B)**. Overexpression of a replication initiation protein or its regulators is a common approach used to test for an effect on initiation [14,39–42]. We found that the replication profile of WT compared to Δ*ccrZ* were very similar in the averaged data **(Fig 2B)** and in the individual replicates **(S2 Fig)**. We do however find an increase in replication at the origin for the overexpression strain, with a dramatic decrease in replication towards the terminus. These data suggest that overexpression of *ccrZ* results in over-initiation of DNA replication further supporting the model that CcrZ acts to regulate replication initiation. We note that prior work measured a decrease in replication initiation for the deletion strain were performed at 37°C under different growth conditions using a different assay [26,27]. Although the difference in our results relative to prior work are unclear, we suggest that it could be due to differences in growth conditions or method of detection.

### CcrZ interacts with DnaA and DnaB

Previously published work and the results presented here suggest that CcrZ regulates replication initiation [26,27]. Misregulation of the replication initiator protein DnaA results in asynchronous initiation and cell elongation [11]. A previous study identified a conditional lethal phenotype with *dnaA1(ts)* Δ*yabA* due to hyper-initiation [27]. In this study, mutations were found in *ccrZ* to suppress the *dnaA1(ts)* Δ*yabA* phenotype further demonstrating a connection between *ccrZ* and replication initiation in *B*. *subtilis* [27]. We therefore tested Δ*ccrZ* in conjunction with *yabA*::*cat* using a spot titer assay to DNA damage **(Fig 2C)**. We found that disruption of the *yabA* gene causes cells to be very sensitive to DNA damage. This result suggests that over-initiation in the *yabA*::*cat* cells [11] is further exacerbated by DNA damage resulting in greater growth interference than Δ*ccrZ* alone. When we combine the *yabA* null with Δ*ccrZ*, we observe the single Δ*ccrZ* phenotype suggesting genetic interaction. To test for a protein interaction, we performed a bacterial two-hybrid with YabA and CcrZ. We did not detect an interaction between YabA and CcrZ which is the same result observed with the *S*. *pneumoniae* proteins [26] **(Fig 2C)**.

To further investigate a role for CcrZ in replication initiation, we tested CcrZ in a bacterial two-hybrid assay with full-length DnaA [9,43,44]. Prior work in *S*. *pneumoniae* did not detect an interaction between CcrZ and full-length DnaA [26]. We also did not detect an interaction between *B*. *subtilis* CcrZ and full-length DnaA **(S1D Fig)**. It has been shown previously that interactions between DnaA and its binding partners are often masked in the full-length protein, yet readily observable when DnaA is divided into its constituent domains [9]. Therefore, we tested CcrZ against DnaA domains I and III and identified an interaction between CcrZ and both DnaA domains for some of the two-hybrid constructs **(Fig 2D)**. Next, we asked if CcrZ interacts with replication initiation proteins DnaB or DnaD again using a bacterial two-hybrid approach as we have done previously [9]. We did not detect an interaction between CcrZ and DnaD **(S1D Fig),** but we did detect an interaction between CcrZ and DnaB, suggesting CcrZ and DnaB could bind in *B*. *subtilis* **(Fig 2D).** The *dnaA*, *dnaB*, and *dnaD* genes are essential and therefore deletions cannot be tested in conjunction with Δ*ccrZ* [45]. We conclude that YabA and CcrZ function in the same pathway and that CcrZ interacts with DnaA and DnaB. Our results showing that CcrZ interacts with DnaA and DnaB could contribute to the replication origin localization shown for CcrZ in *pneumococcal* cells [26].

### Overall structure of CcrZ reveals a pronounced cleft important for function *in vivo*

**Fig 3:**
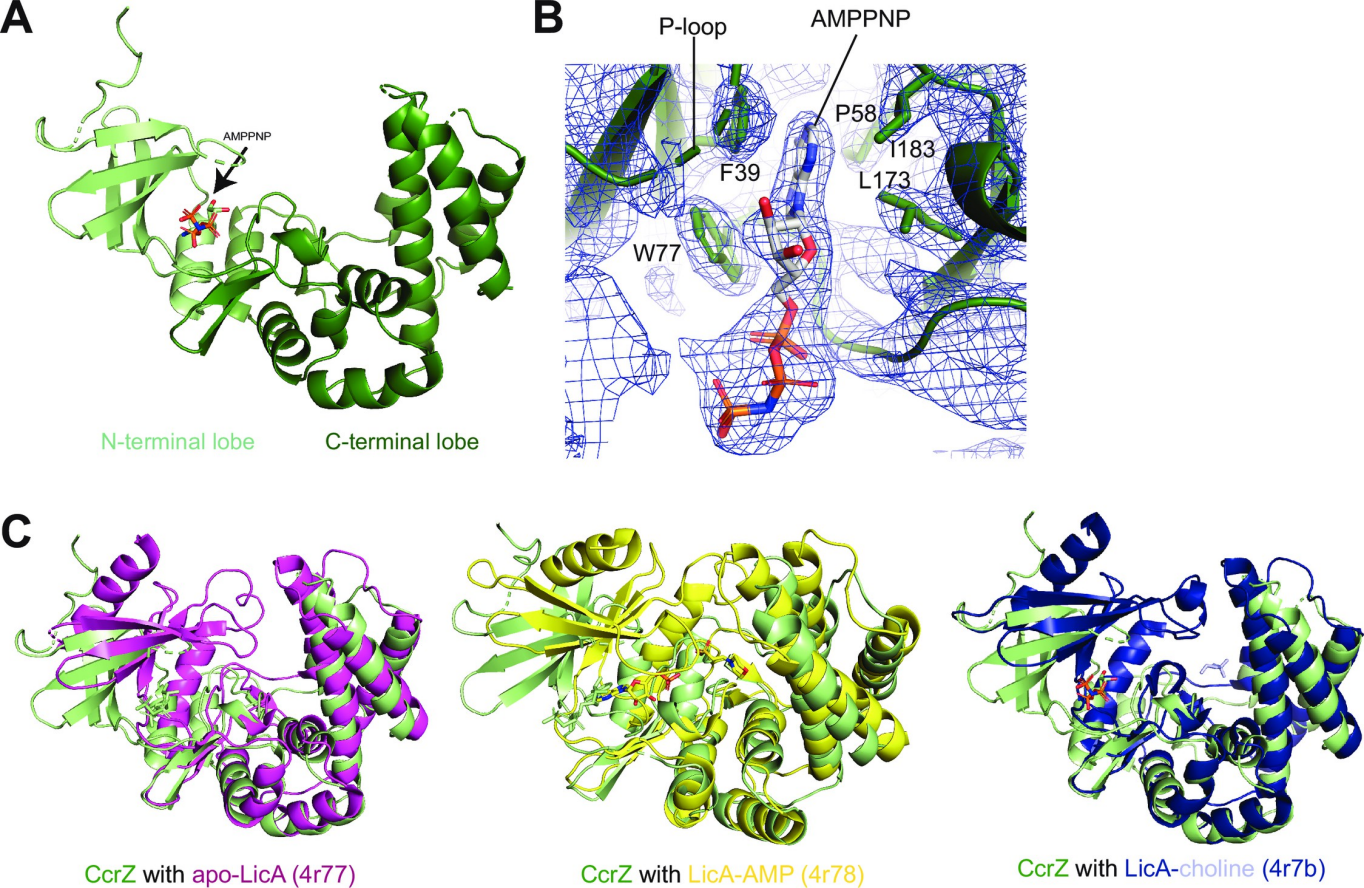
Structural determination of CcrZ and comparison to *S*. *pneumoniae* LicA. **(A)** Structure of CcrZ bound to AMP-PNP. N-terminal lobe in light green, C-terminal lobe in dark green. **(B)** 2F_o_-F_c_ map contoured at 1σ around the AMP-PNP molecule in the kinase pocket (P-loop highlighted) of the CcrZ crystal structure with aromatic and hydrophobic residues flanking the base shown as sticks. While the base occupies its pocket in the enzyme, the rest of the AMP-PNP is swung out of the active site and is directed towards the solvent. **(C)** CcrZ-AMP-PNP aligned with apo-LicA 4r77 (left), CcrZ-AMP-PNP aligned with LicA-AMP 4r78 (middle), CcrZ-AMP-PNP aligned with LicA-choline 4r7b (right). In all three alignments, the cleft between the N- and C-terminal lobes is wider in the CcrZ structure than in the LicA structure.

To further understand the contribution of CcrZ to cell cycle regulation, we solved the crystal structure of *B*. *subtilis* CcrZ bound to AMP-PNP **(Table 1)**. The overall three-dimensional structure of CcrZ resembles that of LicA choline kinase in *S*. *pneumoniae* and other related kinases, consisting of a bilobal structure with a cleft between the two lobes for binding ATP and substrate **(Fig 3A)**. A search for structural homologs using the DALI server revealed apo LicA as the top hit [46] (Z-score: 18.8, rmsd: 3.7 Å) [47] **(S3A Fig)**. Structural alignment of the CcrZ-AMP-PNP structure with LicA confirmed the conservation of an N-terminal lobe (aa 1–79 in CcrZ) containing the P-loop (^22^GGATG^26^ based on alignment with LicA) and other residues important for ATP-binding and kinase activity, and a C-terminal lobe (aa 80–268 in CcrZ) harboring the putative substrate-binding pocket **(Fig 3A and 3B)**. However, in contrast to LicA, the cleft between the N- and C-terminal lobes is wider in the CcrZ-AMP-PNP structure, which could provide insights into how choline kinase activity may be lost in CcrZ **(Figs 3C and S3B)**. This structural difference is likely not attributable to the different stages of catalysis in which LicA and CcrZ were captured, as the width of the cleft is comparable between apo, choline-bound, and AMP-MES-bound LicA structures [46] **(Fig 3C)**. Extending this structural homology to CcrZ, we would predict that its interlobal cleft would remain wide (i.e., wider than substrate-bound LicA) upon substrate binding.

**Table 1:** Data collection and refinement statistics for SelenoMet-derivatized CcrZ-AMP-PNP crystals.

	CcrZ-AMP-PNP selenomethionine derivative
**Data collection**	
Space group	P 43 21 2
Cell dimensions	
*a*, *b*, *c* (Å)	98.118, 98.118, 69.127
α, β, γ (°)	90 90 90
Wavelength (Å)	0.97911
Resolution (Å)	98.12–2.60 (2.72–2.60)
*R*_merge_	0.146 (1.598)
CC_1/2_	0.988 (0.885)
*I* / σ*I*	14.8 (2.6)
Overall Completeness (%)	100 (100.00)
Anomalous Completeness (%)	100 (100.00)
Redundancy	25.5 (27.2)
**Refinement**	
Resolution (Å)	34.69–2.60 (2.72–2.60)
No. reflections	10750 (1139)
*R*_work_ / *R*_free_	0.2418 (0.3190) / 0.2615 (0.3688)
No. atoms	2101
Protein	2068
Ligand	31
Water	2
*B*-factors	82.91
Protein	82.79
Water	87.36
R.m.s deviations	
Bond lengths (Å)	0.008
Bond angles (°)	1.061
Ramachandran favored (%)	93.09
Ramachandran allowed (%)	6.91
Ramachandran outliers (%)	0.00

Structure was solved with data from a single SelenoMet derivative. Values in parentheses are for highest-resolution shell.

**Fig 4:**
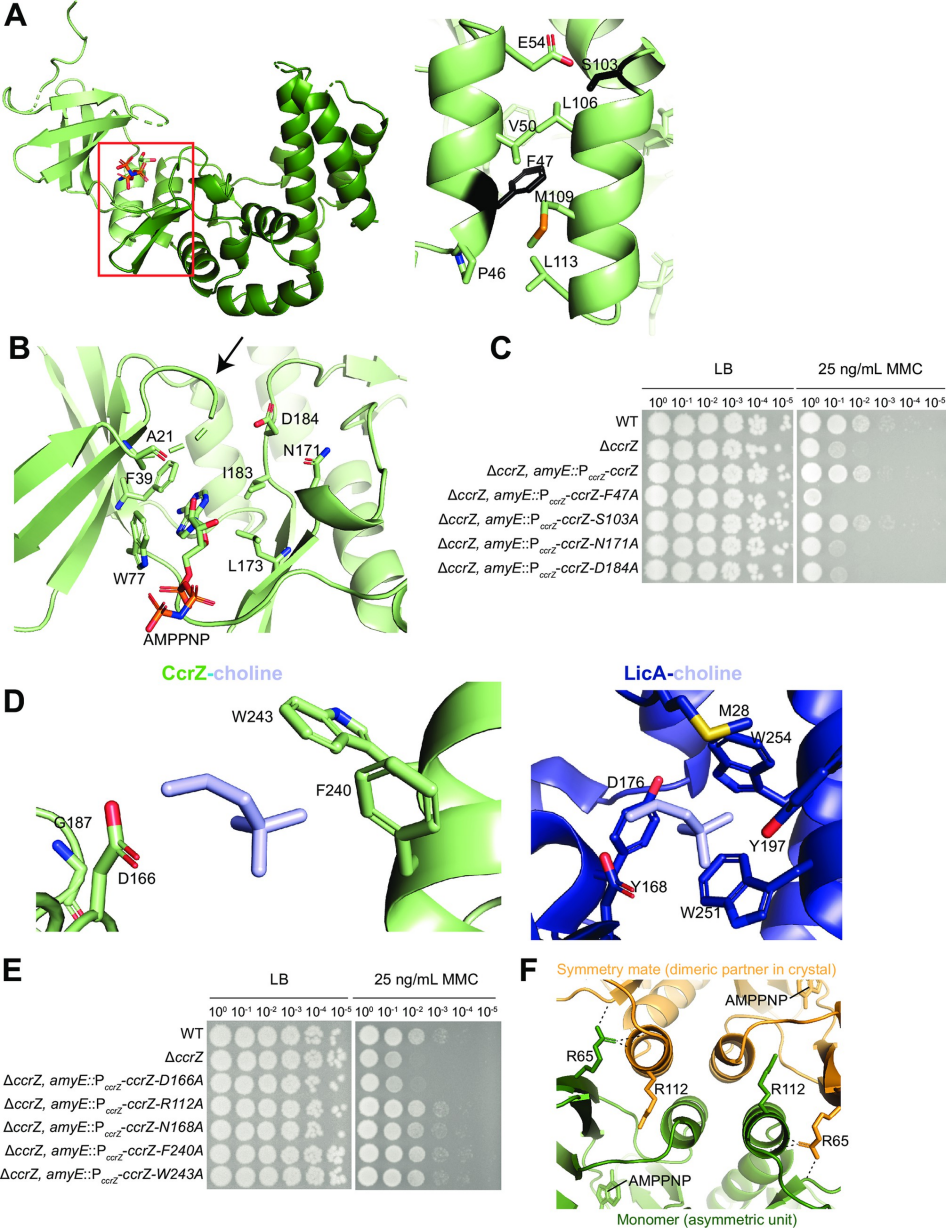
Functional assessment of CcrZ structural features. **(A)** Interaction between two alpha-helices from the N and C terminal lobes of CcrZ. Position in overall structure (left) and residues within the helices (right). F47 and S103 are shown in black. **(B)** CcrZ P-loop (arrow) and catalytic residues highlighted. **(C)** Alanine substitutions of residues in the alpha-helix and catalytic site abolish activity *in vivo*. **(D)** Hydrophobic binding pocket in LicA-choline (right) and CcrZ with choline overlaid (left). Hydrophobic caging residues in LicA and corresponding residues in CcrZ are annotated. **(E)** Alanine substitutions of residues within the binding pocket of CcrZ do not abolish activity *in vivo*. **(F)** Cartoon representation of the dimeric interface in the crystal with AMP-PNP; R112 and R65 on each monomer shown in sticks. H-bonds formed by the side chain of R65 of one monomer with the protein backbone of the other monomer in the crystal are shown as dashed lines.

We observed a unique second alpha-helix in the CcrZ structure that is not present in LicA **(Fig 4A)** and as such, we queried the importance of the interactions that hold the two lobes to maintain the structure of the cleft. This interface is mediated by interactions between two helices, one from each lobe **(Fig 4A)**. We disrupted this hydrophobic interface with a *ccrZ F47A* mutant, which was introduced in a Δ*ccrZ* construct driven by the endogenous *ccrZ* promoter and evaluated its ability to rescue the Δ*ccrZ* phenotype on DNA damage. Consistent with the importance of maintaining the proper relative orientation of the two CcrZ lobes, mutation of F47 failed to complement a *ccrZ* deletion **(Fig 4C),** highlighting the importance of hydrophobic interactions in maintaining this interface. To ensure that the F47A mutant protein was still intact, we performed a Western blot using polyclonal antibodies against CcrZ and observed WT-like accumulation of full-length protein **(S4A Fig)**. CcrZ containing an alanine substitution of S103 (a hydrophilic serine residue at the end of the interface helix on the C-terminal lobe) was able to complement a *ccrZ* deletion **(Fig 4C)**. Our analysis of the overall structure of CcrZ suggests that its domain composition is like that of other kinases including *S*. *pneumoniae* LicA but is characterized by a larger inter-lobe cleft.

### ATP-Mg^2+^-binding pocket of CcrZ

Inspection of the AMP-PNP in the CcrZ structure revealed that the adenine base is sandwiched between hydrophobic/aromatic residues lining the bilobal cleft (F39 and W77 of the N-terminal lobe, and L173 and I183 of the C-terminal lobe) like the LicA-AMP-MES structure [46] **(Fig 3B)**. However, in contrast to the nucleotide-bound LicA structure, the orientation of AMP-PNP is in the opposite orientation in the CcrZ structure. Instead of occupying the active site, the AMP-PNP moiety is rotated such that the sugar and phosphates are facing out of the active site despite the placement of the adenine base in its pocket within the enzyme **(Figs 3B and 4B).** In agreement with a non-canonical AMP-PNP engagement in the kinase site of CcrZ, several P-loop residues of CcrZ remain unresolved in the electron density **(Figs 3B and 4B)**. We propose that our structure captures a state of the enzyme between its apo and substrate-bound forms. By binding ATP in a non-catalytic state, the enzyme enriches this co-substrate in the vicinity of the active site while preventing futile hydrolysis in the absence of the phosphorylation substrate in the active site. The binding of the CcrZ substrate would likely allow the locally concentrated ATP to occupy its pocket in the active site to trigger catalysis. Such induced-fit conformational changes are well documented for other kinases, including the classical example of hexokinases that participate in glycolysis [48].

It was reported that *ccrZ A21V* restored growth in the synthetically lethal *dnaA1(ts)* Δ*yabA* background [27]. In the spot titer assay, A21V appears like WT, indicating that the valine substitution does not disrupt CcrZ function to DNA damage *in vivo* **(S4B Fig)**. As A21 immediately precedes the P-loop of CcrZ **(Figs 4B and S4B)**, we suggest that A21V does not disrupt the ATP binding ability. Although we did not observe electron density suggestive of Mg^2+^, putative divalent metal ion-binding residues are conserved in CcrZ. Specifically, CcrZ N171 and D184 are expected to bind Mg^2+^ to facilitate phosphoryl group transfer during CcrZ-mediated catalysis **(Fig 4B)**. We introduced alanine substitutions at CcrZ N171 and D184 in constructs driven by the endogenous *ccrZ* promoter and evaluated their ability to complement Δ*ccrZ*. We show that these mutants fail to complement the Δ*ccrZ* DNA damage phenotype, strongly suggesting that these mutations abrogate Mg^2+^-mediated kinase activity of CcrZ *in vivo* **(Fig 4C)**. We again observed WT accumulation of N171A and D184A using a Western blot **(S4A Fig)**, suggesting that the mutations do not disrupt protein stability *in vivo*. These observations suggest that the kinase active site of CcrZ that binds ATP-Mg^2+^ is like that of LicA.

### The choline kinase binding pocket is lost in CcrZ

To gain more insights into substrate binding, we focused on the substrate-binding site of the LicA and CcrZ structures. The choline-binding pocket of LicA consists of residues M28, Y197, W251, W254, and Y268 that form a highly hydrophobic and aromatic cage around the quaternary ammonium moiety of choline **(Fig 4D)**. The phosphorylated hydroxyl group of choline hydrogen bonds with LicA D176. Inspection of the analogous region in the CcrZ structure reveals major changes in the substrate-binding pocket that explain the loss of choline kinase activity of this enzyme. Almost all of the bulky side-chains that form the cage surrounding choline in LicA are replaced by smaller residues in the CcrZ structure. Specifically, LicA residues M28, Y197, W251, and Y268 are substituted in CcrZ by A24, G187, F240, and A256, respectively **(Fig 4D)**. An exception is LicA W254, which is retained as tryptophan W243 in CcrZ. The larger cleft between the N- and C-terminal lobes combined with the presence of smaller amino acids in the substrate-binding pocket suggest that the substrate for CcrZ could be larger in dimension than choline. We introduced alanine substitutions at CcrZ F240 and W243 and performed the endogenous *ccrZ* promoter-driven gene complementation spot titer assay to ask how they impact CcrZ function *in vivo*. We included N168A, which changes a residue that is close to the substrate-binding site but not involved in directly binding choline in the LicA structure, as a negative control. None of the mutations disrupted CcrZ function, suggesting that single mutations in its putative substrate-binding pocket do not substantially impact CcrZ function *in vivo* **(Fig 4E)**. Interestingly, LicA D176 is conserved in CcrZ (D166), consistent with the function of this residue in bonding to the phosphorylated hydroxyl group, which is expected to be present in the putative substrate of CcrZ **(S5A Fig)**. Mutation of CcrZ D166 to alanine abolished complementation of CcrZ function in the deletion background, suggesting that kinase activity is required for CcrZ to function *in vivo* **(Fig 4E)**. Using a Western blot with anti-CcrZ, we detected expression of D166A driven by the native promoter inserted at *amyE* **(S4A Fig).** These data indicate that alanine substitution of the potential phosphotransferase residue disrupts protein function and not expression or stability *in vivo*. These observations suggest a major restructuring of the substrate-binding site of CcrZ relative to the choline-binding pocket of LicA that is likely relevant to its adaptation to phosphorylate a different substrate.

### Other unique structural features of CcrZ important for function *in vivo*

Other differences between LicA and CcrZ structures include an N-terminal helix that is absent in CcrZ and a helix in CcrZ (K104-L113) that is absent in LicA. Interestingly, the CcrZ-specific helix forms crystal contacts with another CcrZ molecule (symmetry mate) in the crystal lattice in addition to facilitating the inter-helical contact with the N-terminal lobe **(Fig 4F and 4A, left**). An amino acid side-chain that is critical for this crystal contact is R112, which forms hydrogen bonds with both the side-chain hydroxyl group and the main-chain carbonyl group of S44 of the symmetry mate. We introduced R112A in our gene-complementation assay, which showed that expression of this mutant driven from the endogenous *ccrZ* promoter was sufficient to restore WT-like growth **(Fig 4E)**. Moreover, size-exclusion analysis of purified CcrZ obtained recombinantly from *E*. *coli* suggests that CcrZ protein is a monomer in solution **(S5B Fig)**. Together, these data indicate that the homodimeric interface observed in the crystal lattice is likely a crystallization artifact although it may be suggestive of latent CcrZ protein-binding surfaces that are involved in binding other protein partners *in vivo*. It is interesting that the recent suppressor screen showed that *ccrZ* R65P restored growth in the synthetically lethal *dnaA1(ts)* Δ*yabA* [27]. Inspection of the CcrZ structure reveals that while R65 is a surface-exposed residue that is distant from the catalytic site of the enzyme, it is involved in making multiple hydrogen bonding interactions with the CcrZ-specific helix of the symmetry mate in the crystal lattice **(Fig 4F)**. Using the gene complementation assay, we demonstrate that *R65P* disrupts *ccrZ* function in *B*. *subtilis* **(S4B Fig)**. However, using anti-CcrZ antibodies, we could not detect full length R65P using a Western blot **(S4A Fig),** suggesting R65P destabilizes the native structure of CcrZ protein, which explain the previous suppression data and the lack of *in vivo* complementation of Δ*ccrZ*.

### Ribose stimulates CcrZ kinase activity

**Fig 5:**
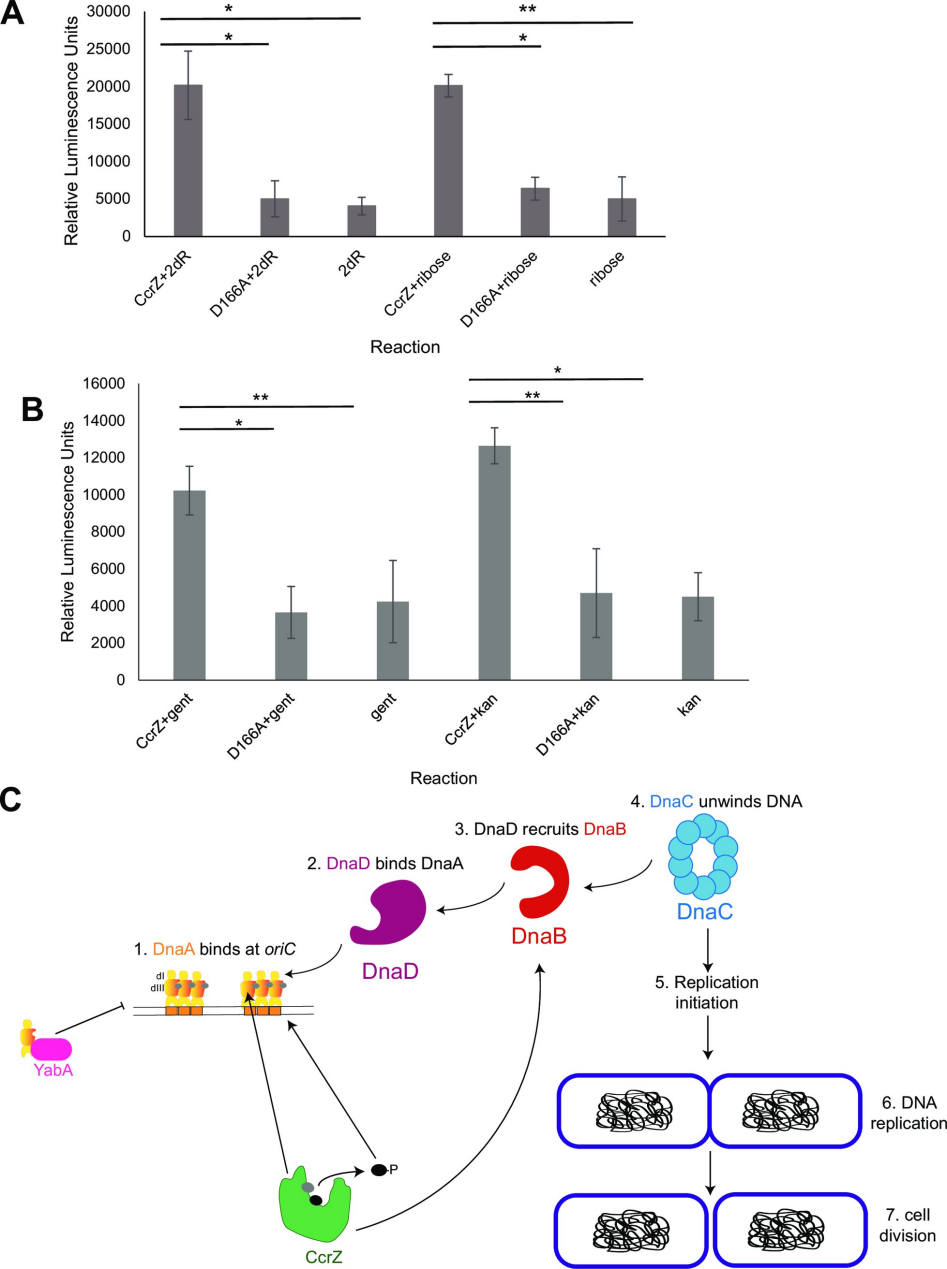
CcrZ demonstrates activity on ribose. **(A)** ATPase assay of CcrZ with D-ribose and 2-deoxy-ribose (2dR). Reactions were performed in triplicate, t-tests were used to assess statistical significance as follows (*p = 0.025–0.03, **p = 8.0E^-5^). Error bars show standard deviation of the mean. **(B)** ATPase assay of CcrZ with kanamycin and gentamicin. Reactions were also performed in triplicate, with t-tests to assess statistical significance (*p = 0.01–0.029, **p = 4.0–6.0E^-4^). Error bars represent the standard deviation of the mean. **(C)** Summary model for CcrZ function in *B*. *subtilis*. CcrZ interacts with DnaA domains I, III and DnaB, perhaps to stimulate replication initiation or help localize CcrZ to the origin. We hypothesize that CcrZ contributes to replication initiation through direct protein-protein interactions and through phosphorylation of a second messenger or metabolite. CcrZ phosphorylation of a ribose-like substrate could serve as an upstream signaling molecule to promote replication initiation through *oriC*. When CcrZ is overexpressed, the increase in phosphorylated substrate and interactions with DnaA and DnaB lead to over-initiation, causing cell elongation and activation of the SOS response.

To examine the activity of CcrZ, we purified CcrZ and CcrZ D166A along with *S*. *pneumoniae* LicA as a control for the experiments that follow **(S5B Fig)**. The lack of structurally conserved residues in the substrate binding pocket suggests that choline is not a substrate for *B*. *subtilis* CcrZ **(Fig 4D)**. CcrZ most likely evolved from a choline kinase-like structural scaffold to retain ATP-binding and kinase motifs but adapted to phosphorylate a different substrate. Inspection of structural homologs of CcrZ using the DALI server revealed several kinase families other than choline kinases, including gentamicin resistance protein (Z-score: 18.3, rmsd: 3.7 Å), spectinomycin phosphotransferase (Z-score: 17.0, rmsd: 2.9 Å), macrolide 2’-phosphotransferase (Z-score: 16.1, rmsd: 4.0 Å), aminoglycoside 3’-phosphotransferase (Z-score: 15.0, rmsd: 4.3 Å), and methylthioribose kinase (Z-score: 11.5, rmsd: 4.2 Å) **(S3A Fig)**. Based on homology to other phosphotransferases, we provided CcrZ with a series of substrates and performed an *in vitro* ADP-Glo ATPase assay as a proxy for kinase activity. To begin, we tested for activity on choline and compared ATPase activity between CcrZ with that of *S*. *pneumoniae* LicA. Our results show that CcrZ does not have activity on choline while LicA shows robust activity **(S5C Fig)**. In sharp contrast to its lack of activity with choline **(S5C Fig)**, CcrZ ATPase activity was stimulated in the presence of D-ribose and 2-deoxy-D-ribose at similar levels **(Fig 5A)**. Alanine substitution of the catalytic D166 residue in the phosphotransferase motif abolished ATPase activity, confirming that purified CcrZ is responsible for the activity detected **(Fig 5A)**. CcrZ ATPase activity was also stimulated to some extent with kanamycin and gentamicin **(Fig 5B)**. Although we observed activity on kanamycin and gentamicin, the *ccrZ* deletion does not display sensitivity to either antibiotic on a spot titer assay **(S5D Fig)**, making these substrates unlikely *in vivo* candidates. We conclude that CcrZ evolved from a choline kinase-like structural scaffold to utilize substrates that share chemical features with sugars such as ribose. We speculate that the phosphorylated product could serve as a second messenger that signals to DnaA, DnaB or perhaps another regulator to ensure proper replication initiation timing *in vivo* **(Fig 5C)**.

## Discussion

All cells must be able to coordinate DNA replication with cell division. Resolving the missing links between DNA replication and cell division has been a focus of research in bacteria for decades. The gene formerly known as *ytmP* (renamed *ccrZ* [26]) in *B*. *subtilis* was annotated as a choline kinase due to its low percent identity to LicA choline kinase in *S*. *pneumoniae* [26,27]. In this work, we show that increased *ccrZ* expression causes cell elongation, induction of the SOS response, and over-initiation of DNA replication. With these data and prior work in *S*. *pneumoniae* and *B*. *subtilis* CcrZ [26,27], we suggest that CcrZ is involved in DNA replication initiation and when left unregulated, changes in replication initiation result in sensitivity to DNA damage and changes in cell length following SOS induction.

Our work indicates that CcrZ in *B*. *subtilis* regulates DNA replication initiation perhaps directly though DnaA and/or DnaB. We establish genetic interaction between *ccrZ* and the negative regulator of DNA replication initiation, *yabA*, suggesting that these genes function within the same pathway. In *S*. *pneumoniae*, CcrZ binds FtsZ and localizes with the origin of replication, DnaA, and the Z-ring during cell division, suggesting that CcrZ links replication initiation with cell division [26]. Our finding that CcrZ interacts with replication initiation proteins suggests that the origin localization shown for *S*. *pneumoniae* CcrZ could be mediated by interactions with DnaA and DnaB. Such localization could scaffold CcrZ in proximity to the origin, allowing for localization of CcrZ kinase activity near effector proteins that are regulated by the phosphorylated product.

Our study and prior work did not observe a cell division defect in Δ*ccrZ B*. *subtilis* cells compared with *S*. *pneumoniae* [26]. Because the Z-ring is the scaffold for other divisome proteins, there are many proteins to prevent inappropriate localization of the Z-ring and the mechanism of these proteins differ between rod-shaped and cocci-shaped bacteria including *S*. *pneumoniae*. MapZ is a positive regulator of FtsZ in *S*. *pneumoniae* that forms a ring at midcell and colocalizes with FtsZ in new cells. Each daughter cell has one Z-ring and a third Z-ring forms at midcell [49]. Although MapZ occurs in *S*. *pneumoniae*, it is not well conserved in Firmicutes, underscoring differences in cell division between rod and cocci-shaped bacterial species [49]. Furthermore, cell wall synthesis and cell division are linked and occur through distinct mechanisms in bacteria of different shapes within Firmicutes. In *B*. *subtilis*, peptidoglycan is inserted laterally into the cell wall of rods to elongate cells, while ovococci like *S*. *pneumoniae* elongate from a growth zone at midcell [for review [50]]. It is plausible that the differences observed between CcrZ regulation of cell division in *B*. *subtilis* and *S*. *pneumoniae* are due to intrinsic differences in cell shape and cell shape regulators between these organisms.

In our work, we were able to solve the crystal structure for CcrZ bound to AMP-PNP. Choline does not stimulate ATPase activity of CcrZ **(S5C Fig),** as homology modeling from our structure would suggest. In addition, the hydrophobic residues involved in caging choline within the binding pocket are not conserved, demonstrating that CcrZ has evolved to phosphorylate a different physiological substrate despite adopting an overall structure of choline kinases. In agreement with the structure, alanine substitution of the remaining residues within the pocket do not abolish function *in vivo*. Strikingly, we show strong activity for CcrZ on D-ribose and 2-deoxy-D-ribose, which is dependent on D166 within Brenner’s phosphotransferase motif [46]. Together, these results suggest that CcrZ evolved from a choline kinase to phosphorylate a ribose-like substrate or a substrate that shares similar chemical features. We find it unlikely that D-ribose or deoxy-D-ribose represent the canonical substrate *in vivo*. *B*. *subtilis* contains the major ribokinase RbsK which does not show homology to CcrZ. In addition, it seems unlikely that CcrZ would contribute significantly enough to the D-ribose 5-phosphate pools where the increase in concentration could be used as a DNA replication initiation signal.

The question that remains is how CcrZ regulates replication initiation. We have shown an interaction between CcrZ and DnaB and domains of DnaA using bacterial two-hybrid. We also show that ablation of kinase activity results in a *ccrZ* null phenotype and that *ccrZ* overexpression causes hyperactive initiation. With these results we propose that CcrZ interacts with replication initiation proteins to localize to the origin. CcrZ then phosphorylates a molecule that helps activate DNA replication initiation through the DnaA-dependent origin [26]. Second messengers have been shown in multiple contexts to act as signaling molecules in many bacteria eliciting responses to environmental changes. For example, the alarmone (p)ppGpp is a starvation second messenger involved in regulation of transcription, translation, and DNA replication [51–53]. C-di-AMP has been shown to couple sporulation with DNA integrity status [54,55], and aid in slowing DNA replication to allow time for repair to occur [56,57]. Although c-di-AMP is involved in several processes, the direct cellular targets are unknown. We suggest that the *in vivo* substrate of CcrZ could be a metabolite or second messenger that helps activate DNA replication initiation. Because we detect interactions between CcrZ and DnaA and DnaB, we speculate that these interacting partners are effector candidates for a small molecule or that DnaA and DnaB interact with CcrZ to help tether it to the replication origin aiding in CcrZ’s regulatory contribution to DNA replication initiation.

## Materials and methods

### Bacteriological methods and chemicals

Bacterial strains, primers, and plasmids are listed in S1–S3 Tables. Detailed descriptions of strain and plasmid construction are also available in S1 Text. *Bacillus subtilis* strains were grown in LB (10 g/L NaCl, 10 g/L tryptone, and 5 g/L yeast extract) or S_750_ minimal media with 1% arabinose or 2% glucose consisting of 1x S_750_ salts (diluted from 10x S_750_ salts: 104.7 g/L MOPS, 13.2 g/L, ammonium sulfate, 6.8 g/L monobasic potassium phosphate, pH 7.0 adjusted with potassium hydroxide), 1x metals (diluted from 100x metals: 0.2 M MgCl_2_, 70 mM CaCl_2_, 5 mM MnCl_2_, 0.1 mM ZnCl_2_, 100 μg/mL thiamine-HCl, 2 mM HCl, 0.5 mM FeCl_3_), 0.1% potassium glutamate, 40 μg/mL phenylalanine, 40 μg/mL tryptophan) at 30°C with shaking (200 RPM). Mitomycin C (MMC), methyl methanesulfonate (MMS), ciprofloxacin, cephalexin, and phleomycin were used in the concentrations listed on spot titer plates. Throughout this study, *B*. *subtilis* was selected for using 5 μg/mL chloramphenicol, 0.5 μg/mL erythromycin, and/or 100 μg/mL spectinomycin. Selection of *E*. *coli* BL21 cells for protein purification was performed with 50 μg/mL kanamycin.

### Competency of *B*. *subtilis* and transformation

A single colony of desired transformation strain was inoculated into 2 mL LM medium (LB with 1 M MgSO_4_) in a 14 mL test tube and grown for 3 hours at 37°C. After ∼3 hours, 20 μL of turbid LM medium was used to inoculate 500 mL MD medium (1X PC buffer [10XPC buffer is 107 g/L potassium hydrate phosphate (anhydrous), 174.2 g/L potassium dihydrate phosphate (anhydrous), 10 g/L trisodium citrate (pentahydrate), up to 1 liter H_2_O], 50% [wt/vol] glucose, 10 mg/mL L-tryptophan, 2.2 g/mL ferric ammonium citrate, 100 mg/mL potassium aspartate, 1 M MgSO_4_, 10 mg/mL phenylalanine) and was grown for an additional 5 hours at 37°C. One microliter of genomic DNA or purified plasmid (∼100 ng/mL) was added to MD medium and was incubated for 90 min at 37°C. Aliquots (200 μL) of cells were plated on a medium containing corresponding antibiotic and incubated at 30°C overnight.

### CRISPR/Cas9 genome editing

To create a scarless deletion of *ccrZ*, we used the CRISPR/Cas9 genome editing protocol described previously [29].

### Spot titer assays

*B*. *subtilis* strains were streaked from glycerol stocks onto LB containing the appropriate antibiotics and incubated overnight at 30°C. A single colony was used to inoculate 2 mL LB media in a 14 mL round bottom culture tube and incubated at 30°C with 200 rpm shaking until the OD_600_ reached 0.5–1.0. Cultures were normalized to OD_600_ = 0.5 and serially diluted to 10^−5^ in sterile saline (0.85% NaCl). 5 μL of the dilution series was spotted onto respective plates and incubated at 30°C overnight. Spot titers were performed in biological triplicate. Plates were imaged using transilluminating white light on top of a black background. Lightness and contrast for the entire image was adjusted, when necessary, in Inkscape. On the day of spot titer assay, fresh LB with agar was prepared and cooled to 60°C on a heat block with stirring. Drug and/or xylose were added to 50 mL conical tubes and molten LB with agar was added to up to 50 mL. The conical tubes were mixed gently by hand and poured into sterile rectangular petri dishes.

### Microscopy

Strains were streaked from glycerol stocks onto individual LB plates containing the appropriate antibiotics and incubated overnight at 30°C. The following morning, cells were removed from the plates by washing with 1 mL of S_750_+2% glucose (WT and deletion strains) or S_750_+1% arabinose (overexpression strains). Optical densities were measured, and cell suspensions were normalized to OD_600_ = 1.0 in the same media. 250 μL of culture was subsequently inoculated into 4.75 mL of media (C_f_ = 0.05) in a 50 mL flask. Cultures were incubated at 30°C with shaking (225 RPM) until OD_600_ reached 0.2 and then 0.1% xylose was added to overexpression strains; 100 ng/mL MMC was added for experiments requiring DNA damage. Once OD_600_ reached 0.5–0.7, 300 μL of culture was removed and FM4-64 was added to 2 μg/mL and incubated at room temperature for 5 minutes. Samples were then transferred to 1% agarose pads made of 1x S_750_ salts. Images were captured using an Olympus BX61 microscope as described [58,59]. Cell length histograms were created using ggplot2 in R studio. Shapiro-Wilk tests were performed to assess whether the data between strains were normally distributed. Wilcoxon rank-sum tests were performed to assess statistical significance.

*tagC-GFP* microscopy: WT, *ccrZ*, and overexpression were streaked onto spectinomycin or spectinomycin and chloramphenicol (overexpression) and incubated at 30°C for 16 hours. The following morning, WT and *ccrZ* were plate washed with S_750_+2%glucose, OD_600_ was normalized to 1.0, and normalized culture was inoculated at OD_600_ = 0.1 into 3 mL minimal medium in 15 mL test tubes. The overexpression plate was washed with S_750_+1%arabinose, normalized, and inoculated in the same fashion as the other strains. 0.1% xylose was added before incubation to induce *ccrZ*. All cultures were incubated at 30°C in a 225 RPM rolling rack. Once the cultures reached a density between 0.7–0.9, cells were imaged. RFP was captured at ~300 ms exposure and GFP was captured at ~800 ms. Analysis was performed using ImageJ. A Kruskal-Wallis test was used to determine whether means of GFP positive cells differed according to genotype. Subsequently, paired Wilcoxon tests were performed with a Benjamini-Hochberg correction to determine pairwise significance.

### Bacterial two-hybrid assays

Full-length *dnaA*, *dnaB*, *dnaD*, and *dnaA* domain I and *dnaA* domain III were fused to T25 and T18 fragments as previously described [9]. Full-length *ccrZ* was fused to T25 and T18 fragments within pKNT25, pKT25, pUT18, and pUT18C vectors in this study (see S1 Text). *yabA* was fused to T25 within pKNT25 and *dnaA* was fused to T18 within pUT18 and pUT18C (see S1 Text). Additionally, remaining *dnaA* and *yabA* constructs were described in prior work [9]. 10 ng each of verified plasmid pairs were co-transformed into chemically competent BTH101 *E*. *coli* (Δ*cyaA*) and plated on LB plates containing 50 μg/mL kanamycin, 100 μg/mL ampicillin, and 10 μg/mL streptomycin. Plates were incubated overnight at 37°C. The following morning, the two-hybrid assay was performed as follows: i) single colonies were inoculated into 3 mL LB with 50 μg/mL kanamycin and 100 μg/mL ampicillin and incubated with shaking at 37°C until the OD_600_ reached 0.5–0.7, ii) OD_600_ were normalized to 0.5 in sterile saline and serially diluted to 10^−3^, iii) 5 μL of the 10^−3^ dilution was spotted onto LB plates containing 40 μg/mL X-gal (suspended in DMSO), 0.5 mM IPTG, 50 μg/mL kanamycin, 100 μg/mL ampicillin, and 10 μg/mL streptomycin, iv) plates were covered with aluminum foil to block light and incubated at 30°C for 24 hours, v) plates were incubated at room temperature for an additional 3 days before imaging.

### Protein purification: WT and D166A CcrZ for enzymatic assays

#### Induction and cell harvest

6xHis-*ccrZ* (or 6xHis-*ccrZ-D166A*) was expressed from pE-SUMO-Kan plasmid as follows: a 50 mL overnight of LB with kanamycin in a 150 mL flask was grown at 37°C from a single colony. The following morning, 20 mL of the overnight culture (OD_600_ ~4.0) was used to inoculate 1 L of LB media with kanamycin in a 2.8 L flask (this was performed in duplicate). The cultures were grown at 37°C shaking for 3 hours, and then induced using 0.5 mM IPTG. Cultures were grown for an additional 3 hours before harvesting. Cultures were transferred to 1L centrifuge buckets and centrifuged at 4000 rpm for 15 minutes and resuspended in cold phosphate buffered saline. The slurries were transferred to 50 mL conical tubes and centrifuged again at 4000 rpm for 20 minutes; supernatant was discarded, and pellets were stored at -80°C.

#### Lysis of cells for protein purification

Cells were resuspended in 30 mL nickel A buffer (20 mM Tris-HCl pH 8.0, 1.4 mM 2-mercaptoethanol, 0.4 M NaCl, and 5% glycerol) with 1 protease inhibitor tablet added. Cell resuspensions were lysed on ice for 5 minutes (10 seconds on, 20 seconds off with 70% amplitude) using a sonicator at 4°C. Subsequently, slurries were added to Oakridge tubes and cellular debris was pelleted at 14000 rpm at 4°C for 45 minutes.

#### Nickel column & tag cleavage using SUMO protease

A Ni-NTA column was equilibrated with 50 mL Nickel A buffer. Then, the supernatant was collected and flowed over a NTA column (GE healthcare) and washed with 5 column volumes (CV) Nickel A buffer. Next, 10 CV of 5% nickel B buffer (2.5 mL nickel B: 500 mL nickel A buffer with 10.21 g imidazole + 47.5 mL nickel A buffer) was applied to the column and 16 x 1.0 mL flow through fractions were collected in 1.7 mL microcentrifuge tubes. Absorbances at 280 nm of each fraction was measured on a Nanodrop Lite and fractions with an absorbance >1.5 were pooled into a 50 mL falcon tube for subsequent purification. The 6xHis tag was cleaved using SUMO protease as follows: Addition of dilution buffer (20 mM Tris-HCl pH 8.0, 5% glycerol, 1 mM EDTA, and 1 mM DTT) to the final salt concentration of 100 mM was added to pooled fractions, 1.0 μM SUMO protease was added, and the mixture was incubated at room temperature for 2 hours. 10,000 Da dialysis tubing was pre-soaked in 100 mL milli-Q water for 10–15 minutes. Digested protein suspension was placed into dialysis tubing and dialyzed in a buffer (20 mM Tris-HCl (pH 8.0), 300 mM NaCl, 5% glycerol) at 4°C with stirring overnight. Fractions from the first nickel column, pre-SUMO protease, and post-SUMO protease samples were loaded onto a 12% SDS-polyacrylamide gel and electrophoresed at 150 V for 1 hour. The gel was stained with Coomassie blue followed by destaining with methanol and acetic acid to determine efficiency of tag cleavage from the protein (efficiency in these conditions was near 100%). 35 mL dialysis buffer was used to equilibrate the column before adding crude protein.

Dialyzed protein was filtered and applied to a NTA column and collected. Then 15 mL each of dialysis buffer, 5% nickel B buffer, and 100% nickel B buffer were used to wash the column. Again, fractions were visualized via 12% SDS-PAGE to assess purity. The protein eluted in the 5% nickel B wash but a 50 kDa contaminating band remained. The protein was concentrated and frozen before further purification. A 10000 Da Centricon was equilibrated with 5% nickel B buffer. Protein was loaded and centrifuged at 4000 rpm for 12 minutes at 4°C. Once ~5 mL of protein remained, the Centricon was placed on ice for 30 minutes. Protein was removed from the Centricon tube and placed into a 15 mL falcon tube. 25% final concentration of glycerol was added (if downstream purification was not being performed within 16 hours) and mixed gently until homogenous and lastly, the entire 15 mL falcon tube was flash frozen in liquid nitrogen and stored at -80°C. If downstream purification was performed 16 hours later, 1 mM DTT was added to the ~5 mL of protein and placed at 4°C overnight.

#### Q Sepharose column

Subsequent purification was performed using an ion exchange (Q) sepharose column using buffer A (20 mM Tris-HCl (pH 8.0), 1 mM DTT, and 5% glycerol) and buffer B (500 mL buffer A with 1 M NaCl). An AKTA FPLC was used and buffer A at 1.0 mL/minute for 30 mL was used to equilibrate the attached column. Protein was thawed on ice and diluted from 400 mM to 50 mM salt using buffer A and filtered. Protein was then loaded into the FPLC at 1.0 mL/minute. Once loaded, a gradient setting was used to elute protein from the column with buffer B (5% to 100%) and 2 mL elution fractions were collected using a fractionator. WT CcrZ eluted early in the fractionation within 5% (~150 mM NaCl). The fractions containing protein were pooled, 1 mM DTT was added, and concentrated using a 10000 Da Centricon (equilibrated with buffer A). Once the protein was concentrated to ~0.5 mL, 25% final concentration of glycerol was added, and 10 μL aliquots were dispensed into 1.7 mL microcentrifuge tubes and flash frozen in liquid nitrogen. Aliquots were stored at -80°C.

#### S200 (size exclusion) column

For the S200 column, the buffer C containing 20 mM Tris-HCl (pH 8.0), 150 mM NaCl, and 1 mM DTT was used to equilibrate the column and was used in final purification. Protein was diluted to ~ 7 mL total volume using buffer C, filtered, loaded, and subsequently injected at 0.5 mL/min. Fractions were collected at 0.5 mL/min with 2 mL/fraction. Fractions 34–38 contained monomeric protein. These fractions were pooled (retention volume ~68 mL) concentrated, glycerol was added to 25%, and 10 μL aliquots were frozen as described above.

#### Protein standards to confirm size of monomeric CcrZ

Lyophilized protein standard mix (15–600 kDa) from Sigma-Aldrich was resuspended in 0.8 mL of running buffer and filter sterilized. The entire volume was injected onto an S200 column equilibrated previously with running buffer. Fractions were collected at 0.5 mL/min with 2 mL/fraction. Thyroglobulin bovine eluted first (~670 kDa), followed by γ-globulins from bovine blood (~150 kDa), albumin (chicken egg grade, ~44.3 kDa), then ribonuclease A type I-A from bovine pancreas (~13.7 kDa), and lastly p-aminobenzoic acid (pABA).

### CcrZ purification for crystallization

#### Production of SelenoMet WT CcrZ

pE-SUMO-kan-6xHis-*ccrZ* was introduced into B834 DE3 strain of *E*. *coli* (*met*^-^). A single colony was used to inoculate 5 mL of LB with 25 mg/mL kanamycin and grown at 37°C for 5 hours. The cells were centrifuged at 4000xg for 10 minutes, washed in SelenoMet medium (Molecular Dimensions Limited) and resuspended in 5 mL SelenoMet medium. The full volume of resuspended cells was inoculated into 100 mL SelenoMet medium with 25 μg/mL kanamycin and 1 mL of 250X methionine solution (Molecular Dimensions) and incubated at 37°C overnight. The next morning, the cells were again centrifuged at 4000xg for 10 minutes, washed with SelenoMet medium, resuspended in 5 mL SelenoMet medium, and used to inoculate 1 L SelenoMet medium with 25 μg/mL kanamycin and 60 mg L-selenomethionine in a 2.8 L flask. Cultures were grown until OD_600_ reached ~0.8 and then 0.5 mM IPTG was added to induce expression. Cells were then grown for 4 hours before harvesting.

#### SelenoMet specific purification

One liter of *E*. *coli* pellet was lysed in 30 mL lysis buffer and 2 protease inhibitor tablets for 6.5 minutes (10 seconds on, 20 seconds off) at 75% amplitude. Cellular debris was pelleted as CcrZ for enzymatic assays. The supernatant was applied to a nickel column twice and collected as flowthrough; all other steps of the nickel column before tag cleavage were the same. Dilution buffer (20 mM Tris-HCl (pH 8.0), 5% glycerol, and 1 mM DTT) was added to the final salt concentration of 135 mM to pooled fractions, 0.25 mM SUMO protease was added, and the mixture was incubated at room temperature for 2 hours. Subsequently, the mixture was dialyzed overnight at 4°C in 10,000 MCW dialysis tubing in a buffer containing 20 mM Tris-HCl pH 8.0, 5% glycerol, and 300 mM NaCl. After approximately 16 hours of dialysis, the CcrZ, His-tag, and SUMO protease mixture was filtered. 30 mL of filtered dialysis buffer was used to equilibrate the nickel column. The filtered protein mixture was applied to a column and flowthrough was passed onto the column once more. 20 mL of dialysis buffer was used to wash the column, followed by 20 mL of 5% nickel B (15 mM imidazole), and 15 mL of 100% nickel B (300 mM imidazole). CcrZ eluted in the flowthrough, dialysis wash, and 5% nickel B wash and all fractions were pooled as a result and concentrated to ~7–10 mL. A subsequent sizing column was the final step in purification of CcrZ for crystal screens. Only the fractions corresponding to the central 80% of the elution peak were collected for concentration. No glycerol was added once the protein was pooled and concentrated to 15–20 mg/mL.

#### Introduction of substrate before crystallizing

4 mM AMP-PNP suspended buffer (20 mM Tris-HCl (pH 8), 150 mM NaCl), 2.5 mM D-ribose, 2 mM MgCl_2_, and 50 mM KCl were added to CcrZ (final CcrZ concentration = 15 mg/mL) and incubated on ice for 30 minutes before setting crystal trays.

#### Crystallization

50 μL of well solution containing 30% PEG 6000, 1 M LiCl, 0.1 M NaOAc (pH 5.84) was added to a sitting drop plate. 1 μL of protein mixture was added to 1 μL of well solution. Trays were sealed and incubated at 16°C for 4–7 days before crystal harvesting.

### Cryoprotection of crystals for crystallography

Fresh crystallization buffer was used to harvest crystals. Specifically, the harvesting/cryoprotection solution contained 30% PEG 6000, 1 M LiCl, 0.1 M NaOAc (pH 5.84), 4 mM AMP-PNP, 20 mM Tris HCl (pH 8), 150 mM NaCl, 2.5 mM D-ribose, 2 mM MgCl_2_, 1 mM DTT and 50 mM KCl. Crystals were first transferred into harvest solution before being flash frozen in liquid nitrogen.

#### Data processing, structure determination, and refinement

The structure of CcrZ-AMP-PNP was determined by single-wavelength anomalous diffraction using SelenoMet-CcrZ crystals at a resolution of 2.6 Å. Diffraction data were collected at the LS-CAT beamline 21 ID-D at the Argonne National Laboratory at 12.671 keV and 100 K. Indexing and processing was performed by Dials (CCP4i). Scaling was performed using Aimless (CCP4i). Resulting.mtz files were used in Autosol. Autosol identified 10 seleno-methionine sites in the asymmetric unit with a figure of merit of 0.328. After phase determination and density modification, Autobuild was used for the initial building of the model, which was further improved and refined iteratively using Coot and Phenix-refine, respectively. Density for the AMP-PNP was obvious during the final rounds or model building, at which point this ligand was included in the model building and final refinement steps. Although MgCl_2_ and D-ribose were included in the crystallization conditions, a clear density in the 2F_O_-F_C_ or F_O_-F_C_ maps to justify their inclusion in the model was absent.

### LicA cloning & purification for enzymatic assays

#### Cloning

A gene block of LicA from *Streptococcus pneumoniae* (codon optimized for use in *B*. *subtilis*) was ordered from IDT with flanks to the pE-SUMO-kan vector described above. LicA was assembled into pE-SUMO-kan and used to transform *E*. *coli* MC1061. The plasmid was verified, extracted, and used to transform BL21 for expression (KJW621).

#### Purification

Nickel affinity chromatography and tag cleavage using SUMO protease were performed as with CcrZ. The second step after tag cleavage and dialysis overnight was identical except LicA eluted in the flowthrough and dialysis wash fractions. An ion exchange (Q) step was performed directly after the second nickel affinity step. The protein from flowthrough and dialysis wash fractions (~15 mL) was at 400 mM NaCl and was therefore diluted to 50 mM NaCl using sepharose A buffer. Diluted LicA was mixed gently and filtered before injecting onto the Q column. LicA eluted at 0.55 mM NaCl. Fractions were pooled, concentrated, and a final concentration of 1 mM DTT and 25% glycerol were added. The final concentration of protein was measured using A_280_ and Beer’s law and resulted in 15 mg/mL.

### Visualization of purified proteins

Purified WT CcrZ and LicA were loaded at 100 μM and CcrZ D166A at 50 μM onto a 12% SDS-polyacrylamide gel, electrophoresed in 1X SDS running buffer (25 mM Tris, 50 mM glycine, 0.1% SDS) at 150 V for 60 minutes, stained in Coomassie Brilliant Blue solution (1.25 mM Coomassie Brilliant Blue, 30% methanol, 10% acetic acid) for 4 hours and incubated with shaking in destain (40% methanol, 10% acetic acid) overnight. The following morning, old destain was removed, fresh destain was added, and further incubated with shaking for 4 hours. Subsequently, destain was replaced with water and incubated overnight before imaging.

### ATPase assays

Protein concentrations were calculating using Beer-Lambert’s Law, where A (absorbance) = E*b*c (E = extinction coefficient in reducing conditions, b = path length of light in Nanodrop, c = concentration in M). Substrates (1.25 mM choline, 1.0 mM gentamicin, 0.1 mM kanamycin, 5 mM D-ribose, 5 mM 2-deoxy-ribose) and 0.1 mM pure ATP were incubated in low retention 1.7 mL microfuge tubes with CcrZ or LicA for 30 minutes in a 37°C water bath. Subsequently, the ADP-Glo kit (Promega) was used to deplete unused ATP (40 minutes room temperature incubation to deplete 0.1 mM ATP) and samples were added to a white walled, opaque bottom 96 well plate. Following incubation, ADP resulting from kinase activity was converted back to ATP and used to convert luciferin into light. Relative luminescence units (RLU) were measured on a GloMax (Promega). At least two technical replicates per assay were performed and averaged. t-tests were used to determine if protein with substrate had higher RLU than substrate alone. Bar graphs represent the average RLU per three biological replicates with standard deviation shown.

### Whole genome sequencing

#### Cell growth

WT, KJW117, and KJW119 were restreaked onto solid media and incubated overnight at 30°C. The following morning, they were plate washed with LB, the OD_600_ was normalized to 1.0 and inoculated into 20 mL LB in a 250 mL flask to a final concentration of 0.05 (in triplicate). Flasks were grown in a 30°C water bath with 200 RPM shaking. When KJW119 reached OD_600_ = 0.2, xylose was added to induce expression of *ccrZ* to 0.1%. All cultures were incubated to exponential growth (OD_600_ = 0.65–0.9). An equivalent of OD_600_ = 10.0 of cells was collected from each flask and pelleted at 4,000 x g for 10 minutes. The supernatant was removed and genomic DNA was extracted thereafter. Genomic DNA was sequenced using an Illumina MiSeq (50 cycles).

#### Genomic DNA extraction

Protocol was followed as previously described in [60].

#### Analysis

Reads were aligned to the PY79 genome using Burrows-Wheeler Aligner (BWA), v 0.7.17. Reads with mapping quality lower than 20 were removed from the BAM files using SAMtools (v 1.13). The resulting alignments were sorted by read name using SortSam, and duplicates were removed using MarkDuplicates. The BAM files were then converted to BED format. Consecutive 1-kb window regions were made across the entire length of the genome using makewindows from BEDtools (v 2.29.2). In R, the raw alignment counts were divided by the total number of alignments in millions to control for difference in sample coverage. Subsequently, the counts were log_2_-transformed. 1 kB windows were re-ordered so the origin was present in the middle, with termini on each end. Log_2_-normalized reads were then plotted according to their genomic position. Reads from three biological replicates from each genotype were averaged to create the main text figure.

### Western blotting

#### Growth of cells

Plates were streaked with glycerol stock of respective strain and incubated at 30°C overnight. The following morning, plates were washed with 3 mL LB, and optical density of 1:10 dilution was measured using a spectrophotometer. Plate washed cultures were diluted to an optical density of 1.0 in 6.0 mL with LB. 5 mL of normalized culture was inoculated into 95 mL LB in a 1L flask. Strains were incubated at 30°C with 225 rpm shaking until optical density reached ~0.2. At this density, KJW119 was given 2.5 mL of 10% xylose (0.25% final concentration). Strains continued to grow at 30°C and were collected at an optical density between 0.53–0.80. Duplicates of each strain were grown. Cells were centrifuged at 4,200xg for 10 minutes and supernatant decanted. Pellets were frozen at -20°C.

#### Lysate preparation, blotting, and detection

1 mL of lysis buffer (20 mM Tris HCl pH 8.0, 100 mM NaCl, 1 mM DTT, 5 mM EDTA, 1% SDS, 7M urea) was added to thawed pellets. Once suspended fully, cells were sonicated for 30 seconds at 60 Hz and then boiled for 10 minutes in a 100°C heat block. 1 μL of lysates were used to quantify total protein concentration using a Bradford assay. 250 μL of lysate was mixed with 250 μL 2X SDS loading dye. 40–56 μg of lysate from WT, deletion, and endogenous promoter constructs (KJW1, KJW117, KJW284, KJW860, KJW795, KJW791, KJW793, KJW824) and 0.8 μg of the overexpression construct (KJW119) were loaded into a pre-cast 4–20% Mini-PROTEAN TGX 10-well SDS polyacrylamide gel and electrophoresed for 95 minutes at 120 V. The gel was transferred to nitrocellulose using the Bio-Rad semi-dry turbo transfer system. The nitrocellulose was cut just above the 37 kDa marker (BenchMark protein ladder) and blocked in 5% milk in 1X TBST for >2 hours at 4°C with gentle agitation. A 1/1000 dilution of anti-DnaN in 5% milk in 1X TBST was added to the nitrocellulose section above 37 kDa. A 1/500 dilution of anti-CcrZ (polyclonal antibody from exsanguination bleed of immunized MI-1655 rabbit by Labcorp) in 5% milk in 1X TBST was added to the nitrocellulose section below 37 kDa. Primary antibodies were allowed to incubate overnight on nitrocellulose at 4°C. After 16 hours, nitrocellulose sections were washed in 1X TBST for 4 hours at room temperature with slight agitation and frequent TBST changes. A 1/1500 dilution of goat anti-rabbit (commercially available from LiCor) in 5% milk in 1X TBST was added to nitrocellulose sections and incubated at room temperature for 1 hour with slight agitation. After, the washing protocol was repeated. Blots were imaged on a LiCor with 700 nm and 800 nm channels selected. Contrast of images were adjusted in Adobe Illustrator.

## Supporting information

S1 Fig CcrZ and its role in the DNA damage response. **(A)** Cell length histograms of MMC treated WT (n = 634) and *ccrZ* overexpression (n = 634). Dashed line represents the WT+MMC mean length (5.35 μm) (right). Wilcoxon rank-sum tests were performed on populations to test for significance; *p = 1.44E^-15^–2.2E^-16^ **(B)** Representative micrographs of WT, Δ*ccrZ*, and overexpression with FM4-64 membrane stain and *tagC*::*tagC-GFP*. Scale bar represents 10 μm. **(C)** Western blot with anti-CcrZ in WT lysates with MMC (+) or with vehicle control (-) for two replicates. Δ indicates a *ccrZ* deletion lysate. **(D)** Bacterial two-hybrid assay in *E*. *coli* showing CcrZ does not interact with full length DnaA or DnaD. Zipped is the positive control with empty vector used as the negative control.
(TIF)

S2 Fig Whole genome re-sequencing data of replicates. Three individual biological replicates of WT (top), Δ*ccrZ* (middle), and *ccrZ* overexpression (bottom) whole genome re-sequencing. Replication location is on the x-axis and number of reads is on the y-axis. These data show that the individual replicates are consistent.
(TIF)

S3 Fig CcrZ structural comparisons. **(A)** Protein structures with highest Z-scores in comparison to the CcrZ structure using DALI. Redundant entries were removed. **(B)** CcrZ inter-lobe cleft is wider than LicA. Left: CcrZ-AMPPNP with the inter-lobe measurement between Thr25 and Phe240 as a dashed line (14.2 Å). Middle: Alignment of LicA structures of LicA from [46] (4r77, 4r7b, 4r78). Dashed line is the inter-lobe measurement of LicA-AMP (4r78) representing the distance between Thr29 in the P-loop to Trp251 (11.4 Å). Choline not shown. Right: LicA structures aligned with CcrZ-AMPPNP with the inter-lobe measurements shown as dashed lines. Choline not shown.
(TIF)

S4 Fig Investigation of *ccrZ* suppressors previously identified [26]. **(A)** Immunoblot of *ccrZ* substitution mutants. R65P does not show stable expression. **(B)** A21 (left) and R65 (middle) location in CcrZ structure, substitution mutants (A21V and R65P) expressed in *ccrZ* cells (right).
(TIF)

S5 Fig CcrZ sequence alignment, expected size, and lack of choline kinase activity. **(A)** Alignment of LicA CcrZ with *B*. *subtilis* PY79 CcrZ and *S*. *pneumoniae* CcrZ (left). CcrZ in PY79 (based on [36]) is predicted to have 4 N-terminal residues that CcrZ in *S*. *pneumoniae* lacks. Brenner’s phosphotransferase motif is highlighted in green and choline kinase motif is highlighted in yellow. Asterisks indicate identical residues in all three sequences, colons indicate residues with strongly similar properties, periods indicate residues with weakly similar properties (for more information, visit Clustal Omega FAQs). Alignment of CcrZ from *B*. *subtilis* PY79 with CcrZ from *S*. *pneumoniae*. Asterisks indicate identical residues in all three sequences, colons indicate residues with strongly similar properties, periods indicate residues with weakly similar properties (for more information, visit Clustal Omega FAQs). **(B)** Left: Purified WT CcrZ, D166A CcrZ, and LicA from *Streptococcus pneumoniae* electrophoresed on a 12% denaturing polyacrylamide gel stained with Coomassie blue. Right: Size Exclusion Chromatography output of WT and protein standards. Labels above peaks represent molecular weight in kDa. CcrZ purification (orange) with peak at ~68 mL retention, which is equivalent to a 30 kDa monomer. Thyroglobulin (36.4 mL retention), gamma-globulin (44.7 mL retention), ovalbumin (57.1 mL retention), ribonuclease A (78.6 mL), p-aminobenzoic acid (120.7 mL). **(C)** ADP-glo kinase assay with LicA and CcrZ on choline. Reactions were performed in biological triplicate and pairwise t-tests were performed *p = 3.7–8.3E^-5^. **(D)** Spot titer assay of Δ*ccrZ* on gentamicin and kanamycin showing the deletion is not sensitive to either drug.
(TIF)

S1 Table Strains used in this study. (XLSX)

S2 Table Plasmids used in this study. (XLSX)

S3 Table Primers used in this study. (XLSX)

S1 Data Cell length data used to generate Figs 1A and S1A. (XLSX)

S2 Data TagC-GFP quantification used to generate Figs 2A and S1B. (XLSX)

S1 Text Supplemental materials and methods. (DOCX)
