# Supplementary figures and images for: Structure and kinase activity of bacterial cell cycle regulator CcrZ
Source: PLoS Genet. 2022 May 16;18(5):e1010196. doi: 10.1371/journal.pgen.1010196 (PMC9135335; doi:10.1371/journal.pgen.1010196)

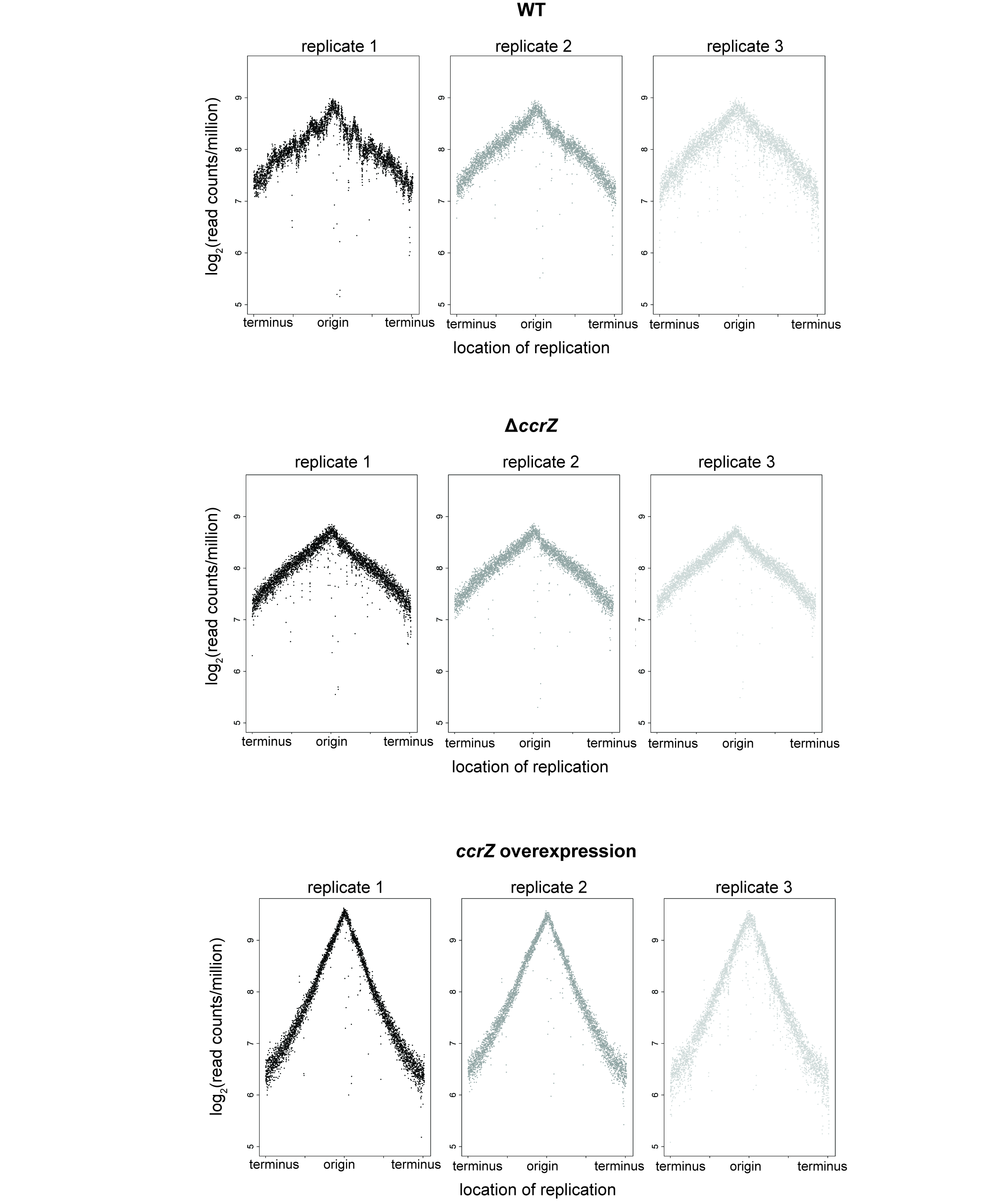

Supplement: S2 Fig — Three individual biological replicates of WT (top), ΔccrZ (middle), and ccrZ overexpression (bottom) whole genome re-sequencing. Replication location is on the x-axis and number of reads is on the y-axis. These data show that the individual replicates are consistent. (TIF) [file pgen.1010196.s002.tif]

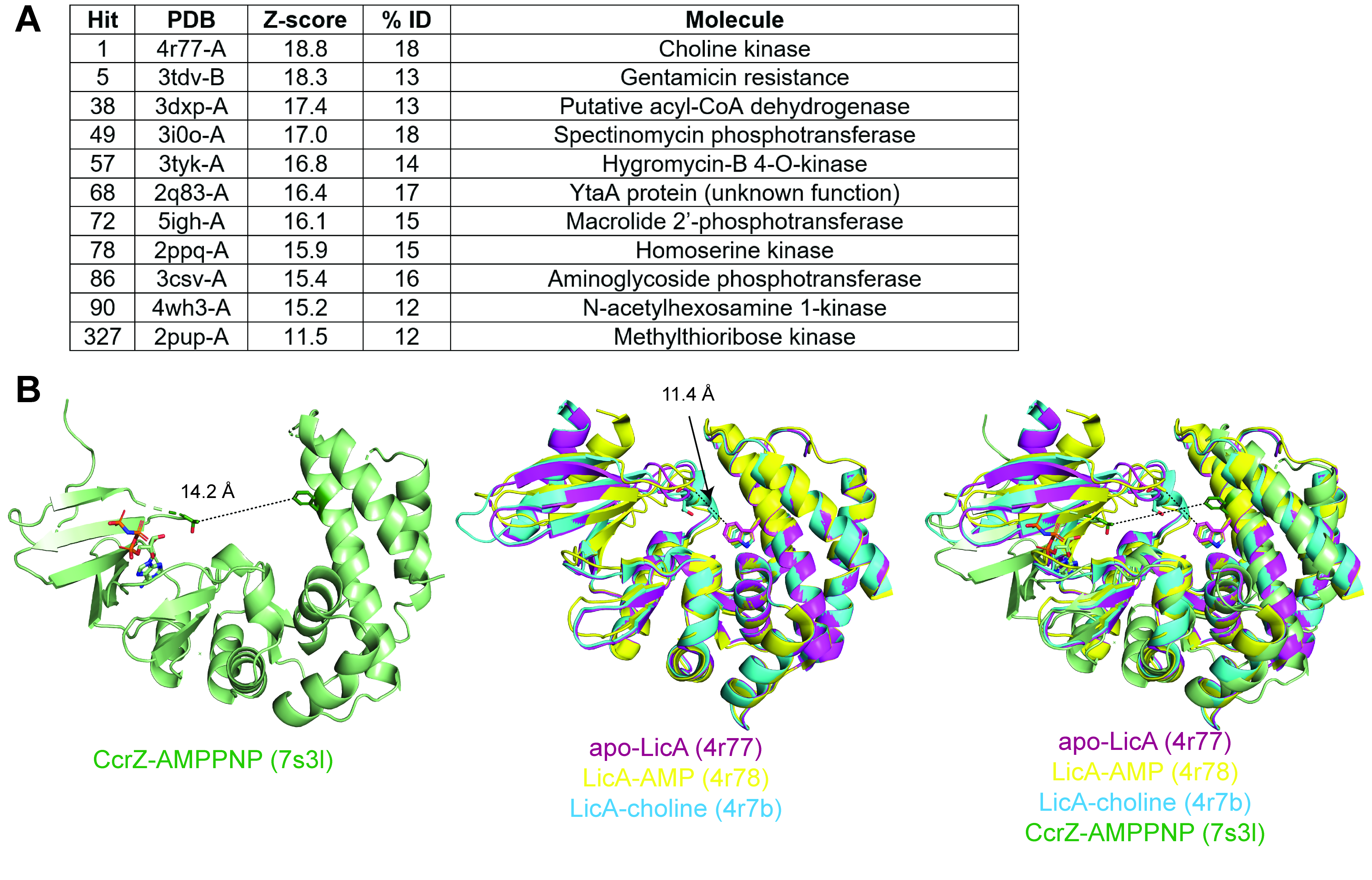

Supplement: S3 Fig — (A) Protein structures with highest Z-scores in comparison to the CcrZ structure using DALI. Redundant entries were removed. (B) CcrZ inter-lobe cleft is wider than LicA. Left: CcrZ-AMPPNP with the inter-lobe measurement between Thr25 and Phe240 as a dashed line (14.2 Å). Middle: Alignment of LicA structures of LicA from [46] (4r77, 4r7b, 4r78). Dashed line is the inter-lobe measurement of LicA-AMP (4r78) representing the distance between Thr29 in the P-loop to Trp251 (11.4 Å). Choline not shown. Right: LicA structures aligned with CcrZ-AMPPNP with the inter-lobe measurements shown as dashed lines. Choline not shown. (TIF) [file pgen.1010196.s003.tif]

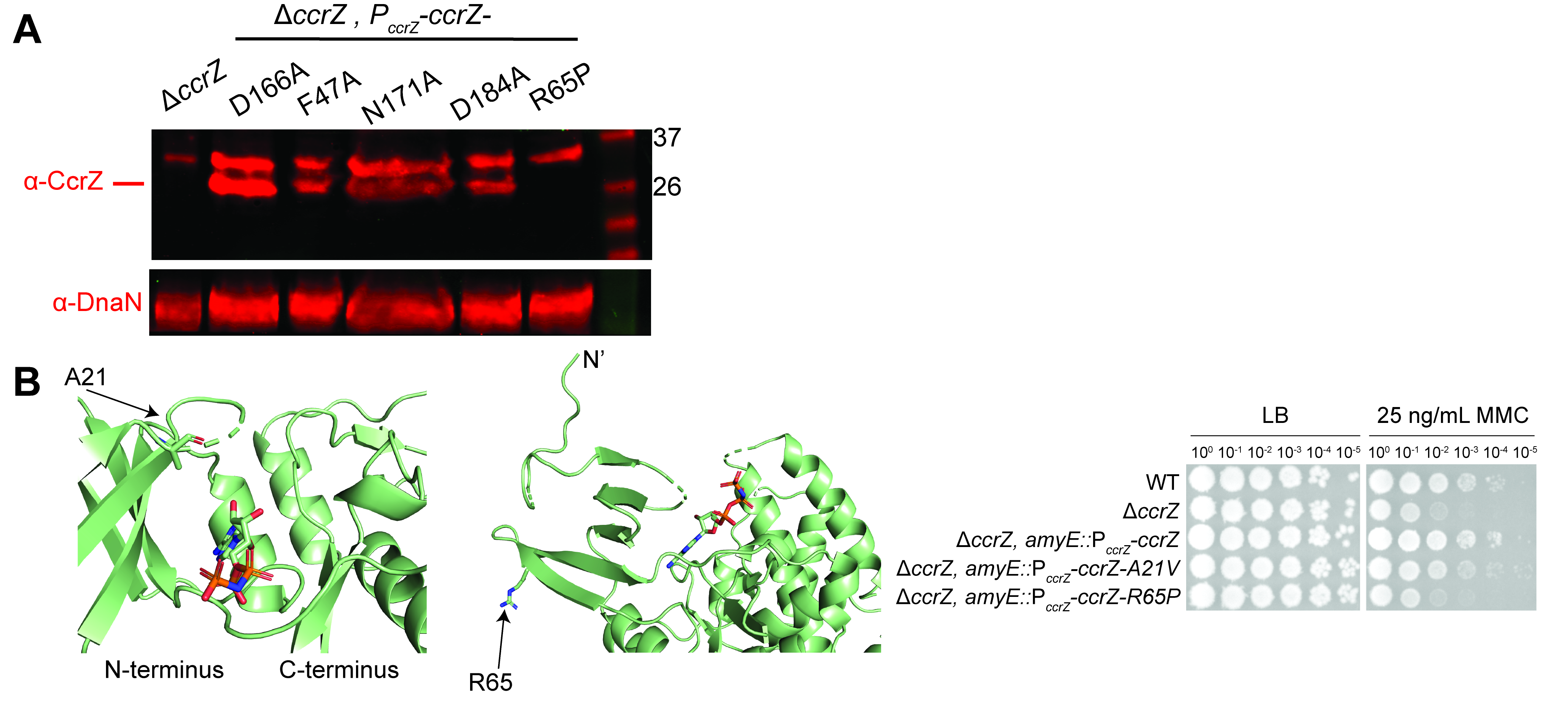

Supplement: S4 Fig — (A) Immunoblot of ccrZ substitution mutants. R65P does not show stable expression. (B) A21 (left) and R65 (middle) location in CcrZ structure, substitution mutants (A21V and R65P) expressed in ccrZ cells (right). (TIF) [file pgen.1010196.s004.tif]

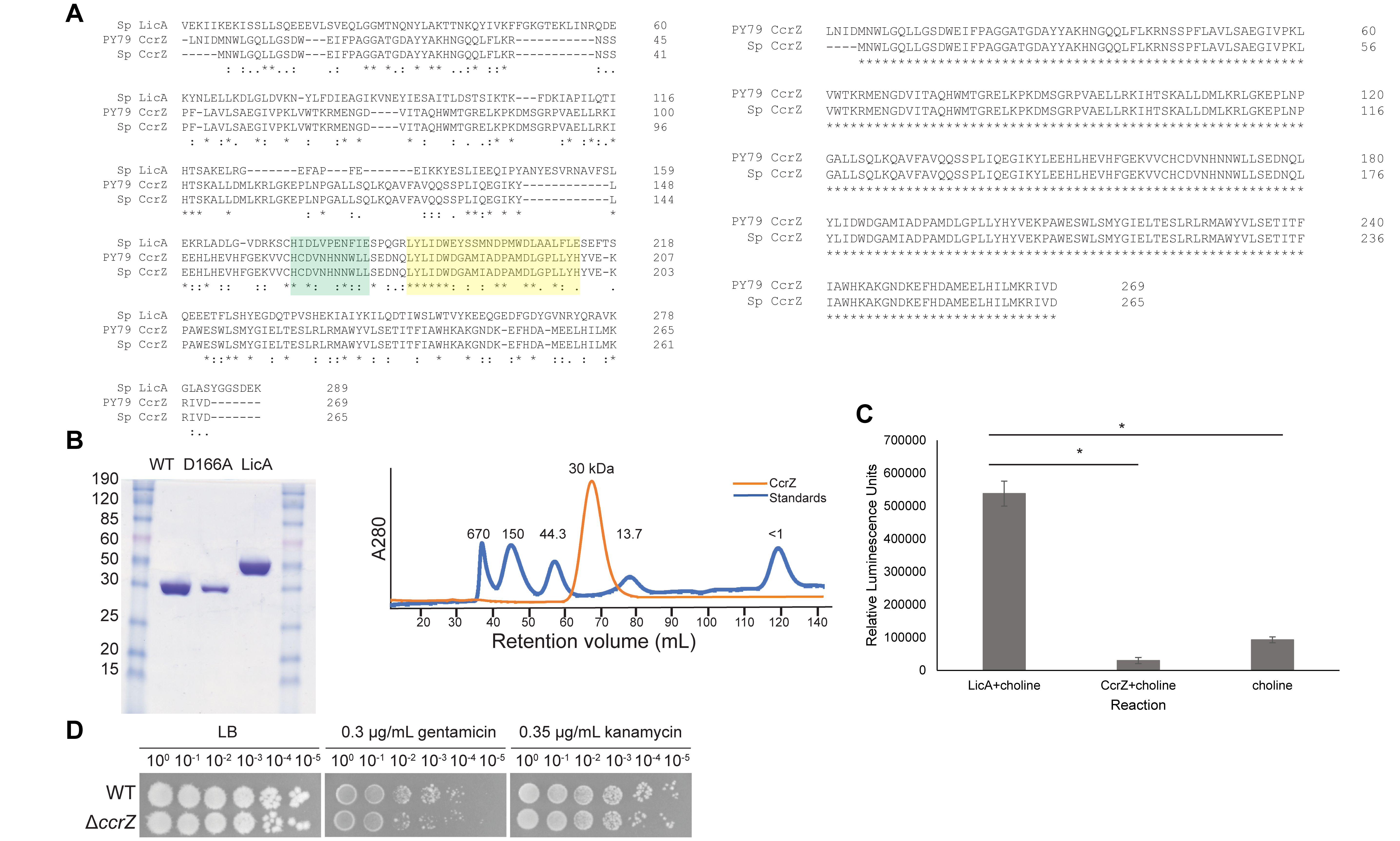

Supplement: S5 Fig — (A) Alignment of LicA CcrZ with B. subtilis PY79 CcrZ and S. pneumoniae CcrZ (left). CcrZ in PY79 (based on [36]) is predicted to have 4 N-terminal residues that CcrZ in S. pneumoniae lacks. Brenner’s phosphotransferase motif is highlighted in green and choline kinase motif is highlighted in yellow. Asterisks indicate identical residues in all three sequences, colons indicate residues with strongly similar properties, periods indicate residues with weakly similar properties (for more information, visit Clustal Omega FAQs). Alignment of CcrZ from B. subtilis PY79 with CcrZ from S. pneumoniae. Asterisks indicate identical residues in all three sequences, colons indicate residues with strongly similar properties, periods indicate residues with weakly similar properties (for more information, visit Clustal Omega FAQs). (B) Left: Purified WT CcrZ, D166A CcrZ, and LicA from Streptococcus pneumoniae electrophoresed on a 12% denaturing polyacrylamide gel stained with Coomassie blue. Right: Size Exclusion Chromatography output of WT and protein standards. Labels above peaks represent molecular weight in kDa. CcrZ purification (orange) with peak at ~68 mL retention, which is equivalent to a 30 kDa monomer. Thyroglobulin (36.4 mL retention), gamma-globulin (44.7 mL retention), ovalbumin (57.1 mL retention), ribonuclease A (78.6 mL), p-aminobenzoic acid (120.7 mL). (C) ADP-glo kinase assay with LicA and CcrZ on choline. Reactions were performed in biological triplicate and pairwise t-tests were performed *p = 3.7–8.3E-5. (D) Spot titer assay of ΔccrZ on gentamicin and kanamycin showing the deletion is not sensitive to either drug. (TIF) [file pgen.1010196.s005.tif]
