# Supplementary material for: Structure and kinase activity of bacterial cell cycle regulator CcrZ
Source: PLoS Genet. 2022 May 16;18(5):e1010196. doi: 10.1371/journal.pgen.1010196 (PMC9135335; doi:10.1371/journal.pgen.1010196)
Supplement: S1 Text — (DOCX) [file pgen.1010196.s011.docx]

**Supporting Text**

**Structure and kinase activity of bacterial cell cycle regulator CcrZ**

Katherine J. Wozniak, Peter E. Burby^#^, Jayakrishnan Nandakumar*, Lyle A. Simmons*

Department of Molecular, Cellular, and Developmental Biology, University of Michigan, Ann Arbor, Michigan, United States of America

* [lasimm@umich.edu](mailto:lasimm@umich.edu)

* jknanda@umich.edu

^#^Current address: Cayman Chemical, Ann Arbor, Michigan, United States of America

**Short title: Crystal structure of cell cycle regulator CcrZ**

**Supporting Materials & Methods**

**Creation of T25 and T18 containing bacterial two-hybrid vectors**

Full-length *ccrZ*, *dnaA*, and *yabA* were PCR amplified with flanks to pUT18C, pUT18, pKNT25, and pKT25. Vectors were linearized using oLM primers. The resulting linearized vectors were joined using Gibson Assembly [1] with the purified insert pieces used to transform *E. coli* MC1061 followed by plating on ampicillin (pUT18C and pUT18) or kanamycin (pKT25 or pKNT25). Transformants were confirmed using colony PCR, the plasmids were isolated using a standard minipreparation procedure, and subsequently sequenced using Sanger to confirm proper construction. Below are the primer combinations to create the gene of interest flanking the given plasmid (oKJW) or amplification of the vector alone (oLM).

- pUT18C-ccrZ: oKJW337+oKJW338
- pUT18-ccrZ: oKJW323+oKJW324
- pKT25-ccrZ: oKJW325+oKJW326
- pKNT25-ccrZ: oKJW327+oKJW328
- pKNT25-yabA: oKJW371+oKJW372
- pUT18-dnaA: oKJW388+oKJW389
- pUT18C-dnaA: oKJW386+oKJW387
- pUT18C: oLM140+oLM141
- pKT25: oLM146+oLM147
- pUT18: oLM217+oLM218
- pKNT25: oLM211+oLM212

**Detailed description of strains created**

**Complementation of** Δ***ccrZ* using the *ccrZ* endogenous promoter (KJW284: *amyE::P_ccrZ_-ccrZ-chl*)**

520 bp upstream of the coding sequence of *ccrZ* was used as the “endogenous promoter.” This upstream region and the entire WT *ccrZ* sequence in PY79 was amplified from genomic DNA using oKJW72+106. Plasmid pPB244 was amplified without the xylose utilization gene but overlapping the endogenous promoter using oKJW10+107 (product termed “*amyE* up without *xylR*”). The *amyE* up fragment without *xylR* was stitched with the endogenous promoter and *ccrZ* using oKJW10+72 followed by Gibson Assembly with the pPB244 backbone. The Gibson Assembly reaction was used to transform *E. coli*, colony PCR verified, and the plasmid was purified. The resulting plasmid (pKJW1) was used to transform *B. subtilis* Δ*ccrZ.* Resulting chloramphenicol resistant colonies were restreaked, and colony PCR verified. The *amyE* locus was subsequently PCR amplified and confirmed using Sanger sequencing. In addition, Sanger sequencing was used to confirm the presence of each substitution incorporated into the *ccrZ* gene that was tested in the assays described in this work*.*

**pE-SUMO-KAN-*ccrZ* in BL21 and B834 DE3 (KJW442, KJW635)**

Overexpression plasmid pE-SUMO-KAN containing 6xHis and a ULP1 SUMO protease cleavage site was obtained from Lindsay Matthews (LAM25) and the backbone was amplified using oLM1+2 [2]. The WT *ccrZ* gene insert was amplified from PY79 genomic DNA using oKJW197+198. Inserts and vector were joined using Gibson Assembly and used to transform MC1061 *E. coli* followed by selection with kanamycin, and colony PCR verified. Plasmids were prepared and used to transform chemically competent BL21 or B834 DE3, with selection on kanamycin. The resulting strains (KJW442, 635) were used to overexpress protein for purification.

**pE-SUMO-KAN-CcrZ-D166A in BL21 (KJW444)**

Plasmid pE-SUMO-KAN containing 6xHis and a ULP1 SUMO protease cleavage site was obtained and amplified as detailed above. The *ccrZ*-*D166A* insert was amplified from KJW280 genomic DNA with oKJW197+198 and joined using Gibson Assembly as detailed above.

**pE-SUMO-kan-LicA in BL21 (KJW612)**

Plasmid pE-SUMO-KAN containing 6xHis and a ULP1 SUMO protease cleavage site was obtained and amplified as detailed above. A codon-optimized gene block of the *licA* sequence from *Streptococcus pneumoniae* R6 was synthesized by IDT. Primers oKJW281 and oKJW282 were used to amplify the *licA* sequence with flanks to pE-SUMO-kan. The resulting amplicon was inserted into the vector using Gibson Assembly and used to transform MC1061. Transformants were selected with kanamycin, followed by verification using standard colony PCR. The plasmid was isolated and used to transform chemically competent BL21, with kanamycin used for selection.

**Creation of alanine substitution mutants in CcrZ (*amyE::P_xyl_ -ccrZ*-chl*)**

*CcrZ D166A, D184A D186A overexpression constructs in* Δ*ccrZ*

Alanine substitutions were created using site directed mutagenesis with primers containing GCA to code for alanine instead of the WT codon. All reactions were performed using Q5 polymerase and products were visualized on 1% agarose gel and either PCR purified, or the specific band was excised from gel and purified using standard procedures.

KJW280 (D166A): oKJW94+98 (“left”) and oKJW96+97 (“right”) on PY79 genomic DNA and left and right were mixed and amplified using oKJW94+96 to create the full *ccrZ*-D166A piece for insertion into pPB244.

KJW282 (D184,186A): oKJW94+100 (“left”) and oKJW96+99 (“right”) on PY79 genomic DNA and amplicons mixed and amplified using oKJW94+96.

Overexpression vector backbone beyond *amyE* flanking sites was amplified using oKJW103+105. The *amyE-xylR-Pxyl* (“*amyE* up”) piece was amplified from pPB244 using oKJW10+93 and *cam-amyE* (“*amyE* down”) downstream piece with flanks to *ccrZ* was amplified using oKJW13+95. Each construct was used in an overlapping PCR reaction amplified with *amyE* up pieces and oKJW10+96. The resulting piece was again used in an overlapping PCR reaction amplified with oKJW10+96 to yield “*amyE-Pxyl-ccrZ*_*D166A* (or D184,186A)-*amyE*.” The resulting PCR product was used in a Gibson Assembly reaction with the pPB244 backbone containing spectinomycin resistance and used to transform MC1061 *E. coli* cells, with selection for chloramphenicol and spectinomycin. Resulting transformants were colony PCR verified, and the plasmids were purified using standard procedures (pKJW2 and pKJW3). Plasmids were then used to transform *B. subtilis* Δ*ccrZ* and with selection for chloramphenicol resistance. Resulting chloramphenicol resistant transformants were restreaked and colony PCR verified. The *amyE* locus was Sanger sequenced to confirm the correct nucleotide substitutions were present in the *ccrZ* gene*.*

**Creation of alanine substitution mutants in *ccrZ* (*amyE*::P_ccrZ-_*ccrZ**-*chl*)**

The *ccrZ* (*F47A, R112A, S103A, D166A, N171A, D184A, N186A, F240A, W243A*) constructs expressed from the endogenous promoter at *amyE* in Δ*ccrZ* were constructed as follows*.*Alanine substitutions in *ccrZ* were created using the same primers as for overexpression (P*xyl*) constructs. The endogenous promoter of *ccrZ* was amplified using oKJW106 and oKJW339. P*ccrZ* and *ccrZ* alanine substitutions were stitched together using oKJW106 and oKJW96 and assembled into the plasmid with *amyE* flanks.

KJW795 (*F47A*): oKJW347+106 (“left”) oKJW346+96 (“right”) on PY79 genomic DNA and left and right were mixed and amplified using oKJW94+96 to create the full *ccrZ*-*F47A* piece for insertion into the modified vector.

KJW750 (*R112A*): oKJW311+106 (“left”) and oKJW312+96 (“right”) on PY79 genomic DNA and left and right were mixed and amplified using oKJW94+96 to create the full *ccrZ*-*R112A* piece for insertion into modified vector.

KJW797 (*S103A*):  oKJW349+106 (“left”) oKJW348+96 (“right”) on PY79 genomic DNA and left and right were mixed and amplified using oKJW94+96 to create the full *ccrZ*-*S103A* piece for insertion into modified vector.

KJW860 (*D166A*): oKJW98+106 (“left”) oKJW97+96 (“right”) on PY79 genomic DNA and left and right were mixed and amplified using oKJW94+96 to create the full *ccrZ*-*D166A* piece for insertion into modified vector.

KJW756 (*N168A*): oKJW313+106 (“left”) and oKJW314+96 (“right”) on PY79 genomic DNA and left and right were mixed and amplified using oKJW94+96 to create the full *ccrZ*-*N168A* piece for insertion into modified vector.

KJW791 (*N171A*): oKJW343+106 (“left”) and oKJW342+96 (“right”) on PY79 genomic DNA and left and right were mixed and amplified using oKJW94+96 to create the full *ccrZ*-*N171A* piece for insertion into modified vector.

KJW793 (*D184A*): oKJW345+106 (“left”) and oKJW344+96 (“right”) on PY79 genomic DNA and left and right were mixed and amplified using oKJW94+96 to create the full *ccrZ*-*D184A* piece for insertion into modified vector.

KJW752 (*F240A*): oKJW315+106 (“left”) and oKJW316+96 (“right”) on PY79 genomic DNA and left and right were mixed and amplified using oKJW94+96 to create the full *ccrZ*-*F240A* piece for insertion into modified vector.

KJW754 (*W243A*): oKJW318+106 (“left”) and oKJW317+96 (“right”) on PY79 genomic DNA and left and right were mixed and amplified using oKJW94+96 to create the full *ccrZ*-*W243A* piece for insertion into modified vector.

**Creation of previously published suppressor mutants in *ccrZ***

KJW799 (*A21V*) [3]: oKJW351+ oKJW106 (“left”) and oKJW350+oKJW96 (“right”) on PY79 genomic DNA and left and right were mixed and amplified using oKJW94+96 to create the full *ccrZ*-*A21V* piece for insertion into modified vector.

KJW824 (*R65P*) [3]: oKJW353+oKJW106 (“left”) and oKJW352+oKJW96 (“right”) on PY79 genomic DNA and left and right were mixed and amplified using oKJW94+96 to create the full *ccrZ*-*R65P* piece for insertion into modified vector.

**Alignment of protein FASTA sequences using Clustal Omega**

Protein BLAST of the CcrZ sequence from *B. subtilis* PY79 against *Streptococcus pneumoniae* produced a top hit with 100% identity within 265 residues and 98% overall and an e-value of 0 (accession: [CJR43657.1](https://www.ncbi.nlm.nih.gov/protein/CJR43657.1?report=genbank&log$=prottop&blast_rank=1&RID=XVKBR3UD016)). These sequences were aligned using Clustal Omega. In addition, both sequences were aligned to LicA from *S. pneumoniae* CP020549.1[4].

**Supporting References**

1. Gibson KJ, Benkovic SJ. Synthesis and application of derivatizable oligonucleotides. Nucleic Acids Res. 1987;15:6455-67.

2. Matthews LA, Simmons LA. Regulation of DNA Binding and High-Order Oligomerization of the DnaB Helicase Loader. J Bacteriol. 2020;202(21). Epub 2020/08/21. doi: 10.1128/JB.00286-20. PubMed PMID: 32817095; PubMed Central PMCID: PMCPMC7549361.

3. Anderson ME, Smith JL, Grossman AD. Multiple mechanisms for overcoming lethal over-initiation of DNA replication. bioRxiv. 2021:2021.05.06.442943. doi: 10.1101/2021.05.06.442943.

4. Wang L, Jiang YL, Zhang JR, Zhou CZ, Chen Y. Structural and enzymatic characterization of the choline kinase LicA from Streptococcus pneumoniae. PLoS One. 2015;10(3):e0120467. Epub 2015/03/18. doi: 10.1371/journal.pone.0120467. PubMed PMID: 25781969; PubMed Central PMCID: PMCPMC4364537.
